# Wildfire Produces Transient Minerals: Speciation, Reactivity, and Fate of Iron and Manganese in Surface Soils Post Wildfire

**DOI:** 10.1021/acs.est.5c07438

**Published:** 2025-12-08

**Authors:** Kyounglim Kang, Elizabeth M. Whelan, Sharon Bone, Mike C. Rowley, Matthew A. Marcus, Jasquelin Peña

**Affiliations:** † Department of Civil and Environmental Engineering, 8789 University of California, Davis, California 95616, United States; ‡ Advanced Light Source, Lawrence Berkeley National Laboratory (LBNL), Berkeley, California 94720, United States; § Department of SoilWater, and Climate, University of Minnesota − Twin Cities, 1991 Upper Buford Cir, St. Paul, Minnesota 55108, United States; ∥ Stanford Synchrotron Radiation Lightsource, 497525 SLAC National Accelerator Laboratory, Menlo Park, California 94025, United States; ⊥ Institute of Bio- and Geosciences: Agrosphere, Forschungszentrum Jülich, Jülich 52428, Germany; # Energy Geosciences Division, Earth and Environmental Sciences Area, Lawrence Berkeley National Laboratory, Berkeley, California 94720, United States; ∇ Department of Geography, University of Zurich, Zurich 8057, Switzerland

**Keywords:** wildfire, fire impacts, soil minerals, micronutrient cycling, iron, manganese, siderophores, hausmannite, bixbyite, maghemite, hematite

## Abstract

Wildfire leads to the deposition of an ash layer at the land surface. This material typically has alkaline properties, abundant pyrogenic carbon, and elevated metal concentrations compared to surface soils. Here, we compare ash and surface soils collected three weeks and two years after the Glass Fire (St. Helena, California, USA) to determine how wildfire affects the speciation and reactivity of iron (Fe) and manganese (Mn), two micronutrients that influence many soil processes. Synchrotron-based analyses revealed that wildfire generated stable Fe oxides like maghemite (γ-Fe_2_O_3_) and hematite (α-Fe_2_O_3_) that likely derived from surface soil or mineral dust rather than the vegetation, and Mn oxides such as hausmannite (Mn_3_O_4_) and bixbyite (Mn_2_O_3_) that derived primarily from aboveground biomass. However, these pyrogenic minerals were absent in soils collected two years after the fire. Their loss from the soil surface may result from a combination of erosion, translocation to deeper soil layers, and chemical weathering. The latter hypothesis is supported by the enhanced mobilization of Fe and Mn from ash upon the addition of pyoverdine, a model biogenic ligand. The increased mobility of micronutrients from fire-derived minerals in ash in response to microbial and root exudation may facilitate postfire soil and vegetation recovery.

## Graphical abstract

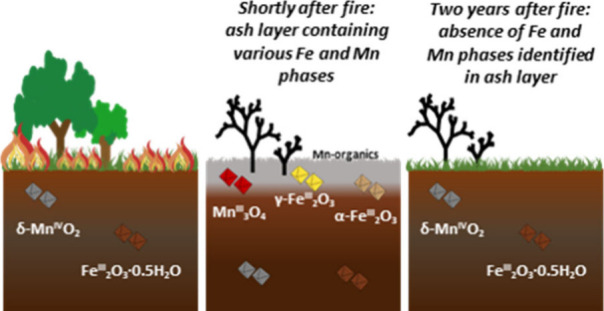

## 1 Introduction

Wildfire is a recurrent ecological process, especially in fire-adapted Mediterranean ecosystems.^1–4^ However, climate change and land management practices have led to an increase in the frequency and severity of individual fires in recent decades,^5–7^ drawing increased attention to the environmental and ecological impacts of fire. One of the most salient consequences of wildfires is the production and deposition of ash at the landscape surface. This ash layer, produced by the burning of vegetation, litter, and topsoil, comprises a heterogeneous mixture of charred organic and mineral material.^8^ Previous research indicates that these pyrogenic residues can alter soil properties,^9–11^ including the elemental composition, pH, water repellency, and nutrient availability.^8,12–14^ However, little data are available regarding the molecular-scale biogeochemistry of minerals within wildfire ash.

The composition of wildfire ash depends on the type of fuel, burn severity, and the burn temperature.^9,15^ At temperatures below 450–500 °C, wildfire ash can contain up to 50% (mass/mass) pyrogenic organic carbon that is enriched in aromatic, polyaromatic, and polycyclic aromatic hydrocarbons.^1,16,17^ As the burn temperature increases (>500 °C), the resulting ash becomes enriched with metals and other inorganic species.^18,19^ The near-complete combustion and mineralization of organic material leads to the formation of carbonate minerals and transformation of base cations into metal oxides.^15,20,21^ The burning of soil minerals at the land surface can also transform secondary soil minerals (e.g., loss of interlayer water in clay minerals and/or dehydroxylation and structural transformation of minerals such as kaolinite and vermiculite^22,23^ ). Trace elements originating from the vegetation, litter, or soil may also condense and crystallize to form new mineral phases. Overall, these inorganic components can comprise as much as 70% (mass/mass) of wildfire ash,^24^ although their structure and reactivity remain largely uncharacterized.

To date, little data is available on the speciation of essential soil micronutrients like Fe and Mn^25,26^ in wildfire impacted systems. For Fe, laboratory studies have shown that heating synthetic ferrihydrite led to formation of maghemite (200–280 °C), magnetite (227–400 °C), and hematite (>400 °C).^27,28^ Similarly, heating A-horizon Ferrosol soil samples above 400 °C caused the transformation of ferrihydrite and goethite to hematite.^29^ For Mn, heating O-horizon soils, which are rich in organic matter (>20%), transformed Mn into Mn­(IV) oxides (e.g., birnessite) at temperatures above 300 °C.^30^ These phases then underwent thermal collapse at temperatures above 400 °C.^30^ Although existing literature indicates that heating can alter the chemical structure of Fe and Mn minerals, field-based data on the speciation of Fe and Mn in ash produced during wildfires remains scarce.^31^

In this study, we examined the speciation and reactivity of Fe and Mn in wildfire ash collected three weeks after the Glass Fire (St. Helena, California), as well as in unburned and burned surface soils that were collected two years after the fire. Samples were characterized in terms of elemental composition using X-ray fluorescence mapping and chemical speciation using bulk and spatially resolved X-ray absorption near edge structure (XANES) spectroscopy. Additionally, water extractions and batch dissolution experiments were conducted to examine the potential to mobilize Fe and Mn in ash-water and soil-water suspensions with and without the addition of pyoverdine, a biogenic ligand with a high affinity for Fe­(III) (log *K*: 44.6 for [Fe­(III)­HPVD]) and Mn­(III) (log *K*: 47.5 for [Mn­(III)­HPVD]).^32^ We selected this compound as a model biogenic ligand that can mobilize Fe and Mn from mineral surfaces and thus promote microbial and vegetation regeneration.^33^ This is the first attempt to characterize the speciation, reactivity, and fate of Fe and Mn phases in wildfire ash. Our results provide insights into postfire micronutrient cycling and its implication for soil resilience and recovery.

## 2 Materials and Methods

### 2.1 Materials

All chemicals were obtained as ACS-grade chemicals (Table S1) except for pyoverdine (C_56_H_88_N_18_O_22_), which was synthesized in-house (Note S1). Ultrapure water (resistivity > 18.2 MΩ·cm, TOC < 2 ppb, Milli-Q, Millipore) was used to prepare all solutions and suspensions.

### 2.2 Site Description and Sample Collection

The Glass Fire, which occurred in Northern California from September 27 to October 20, 2020, burned 67,484 acres across Napa and Sonoma counties, causing extensive damage to not only forested landscapes but also residential area.^34^ Burn severity within the fire perimeter ranged from very high to moderate according to the Monitoring Trends in Burn Severity (MTBS) and field inspection (Figure S1 and Table S2).^34–36^ The sampling sites, which burned between September 29 and October 2, 2020, are located in and around Pride Vineyard in the Mayacamas Mountains (38.526071, −122.563454, 650 m above sea level), on a ridge that runs between the Napa and Sonoma valleys and borders the Bothe-Napa Valley State Park (Note S2).

Ash (*n* = 4), surface soil (*n* = 10), and biomass (*n* = 2) samples were collected from three dominant vegetation types following an elevational gradient, from mixed spruce–fir forest at the toe slope to chaparral at the slope shoulder and oak forest at the slope summit. Samples were also collected from a grassy area at the edge of a water retention pond (riparian zone). Four ash samples were collected on October 24, 2020, which was three weeks after the fire passed the site and prior to any rainfall.^37^ These samples were collected with a trowel and included loose pyrogenic residues above the soil surface, consisting primarily of burned vegetation and litter with some potential contribution from the uppermost surface soil. Six surface soil samples (top 5 cm) were collected two years after the fire from within the burn perimeter (2-year-burned). The 2-year-burned samples were collected from the same locations as the ash sample (Table S3). While these postfire surface soils did not have a visible ash layer (Table S2), the sampling site still exhibited some visible signs of fire, including the presence of burned biomass and darkened surface soil layers. Unburned samples, including four surface soils (top 5 cm; 2-year-unburned) and two vegetation samples (leaves/needles and small stems from manzanita chaparral and Douglas fir trees) were collected two years after the fire in areas outside the Glass Fire perimeter, from locations that were not impacted by the Glass Fire but have similar soil types to those within the burn perimeter. No ash particles were visually identified in the unburned samples, and any potential airborne ash deposition during or after the fire would be minimal relative to the 5 cm sample depth. A complete sample list (Tables S2 and S3) is provided in the Supporting Information.

### 2.3 Chemical Analyses

All ash and soil samples were air-dried, sieved to 2 mm, and stored at 4 °C. Vegetation samples were dried at 60 °C for 3 days and then ground for 3–4 min at 825 rpm in a ball mill (8530 enclosed shatterbox) and stored at 4 °C. All samples were measured in duplicate to ensure analytical reproducibility.

#### 2.3.1 Total Elemental Composition

The elemental composition of all samples was measured by ALS USA lnc. (Reno, Nevada, USA). The ALS method ME-VEG41a was used for ash and vegetation samples, while the method ME-MS41L was used for soil samples. Briefly, samples were digested in 75% aqua regia (nitric and hydrochloric acids in a 1:3 v/v ratio). All elements except Si, Ti, and Zr were quantitated by inductively coupled plasma-atomic emission spectroscopy (ICP-AES) and mass spectrometry (ICP-MS). Due to their resistance to standard acid digestion and tendency to occur in refractory mineral forms (e.g., quartz, rutile, and zircon), Si, Ti, and Zr were analyzed using portable X-ray fluorescence (pXRF) prior to digestion (ALS method pXRF-34). The detection limits for Si, Ti, and Zr were 0.5%, 0.1%, and 5 ppm, respectively. Samples were not furnaced before analysis; thus, the elemental concentrations reported are not corrected for loss on ignition of organic carbon or volatiles. Statistical analyses were carried out using python.

#### 2.3.2 Total Organic and Inorganic Carbon

The total carbon content of all samples was measured with a FlashSmart elemental analyzer. A second sample aliquot was acid fumigated in order to remove any inorganic carbon,^38^ such that the residual carbon content of the sample provided a measure of the organic carbon content. Briefly, ground samples were dried, placed into glass vials, and then fumigated for >24 h with 12 M HCl in a glass desiccator. After fumigation, samples were left to air for 24 h, moistened, and then redried (65 °C), calculating the change in mass to correct data. The fumigated (organic carbon) and nonfumigated (total carbon) were loaded into capsules and measured, correcting for residual humidity.

#### 2.3.3 Chemical Analyses of Water Extracts

Extracts prepared at a 1:2.5 ratio (W/W) of ash/soil in Milli-Q water (18.2 MΩ) at 20 °C (±1 °C) were used to measure the pH, electrical conductivity, and water-extractable dissolved organic carbon (DOC) of the ash and unburned surface soil and litter samples. The extractions were conducted in duplicate using 50 mL conical tubes by adding 1.0 g of sample and 2.5 mL of water. Samples were mixed on an end-over-end rotator for 1 h and then allowed to rest for 30 min. The pH of sample solutions was then measured potentiometrically (electrode: 6.0150.100, Metrohm), waiting approximately 3 min after probe insertion for pH values to stabilize before measurements. Electrical conductivity was measured using a conductivity probe (6.0917.080, Metrohm). The dissolved organic carbon content was analyzed using a Sievers 5310 C laboratory TOC Analyzer.

#### 2.3.4 Bulk XANES Analyses

To investigate the effects of wildfire on the average Fe and Mn speciation in the ash samples, 2-year-unburned and 2-year-burned samples, and reference materials, we collected Fe and Mn K-edge X-ray absorption near edge structure (XANES) spectra, hereafter “bulk” XANES spectra. Ash and 2-year-unburned samples were homogenized individually (i.e., ground to a uniform consistency) and packed into Al sample holders covered with Kapton prior to analysis at 77 K (LN2 cryostat) at Beamline 4-1 of the Stanford Synchrotron Radiation Lightsource (SSRL) using a Si (220) ϕ = 90 monochromator crystal. The incident beam was set to 1 mm in the vertical dimension and detuned by 50% at 7000 eV for Mn and 7600 eV for Fe. Monochromator energies were calibrated using an Fe foil at 7112 eV and a Mn foil at 6539 eV. Iron and manganese K-edge XANES spectra were collected in fluorescence mode using a solid-state passivated implanted planar silicon (PIPS) detector or a Ge detector equipped with Z-1 filters (Cr for Mn and Mn for Fe). Note that the ash and 2-year-unburned samples were analyzed at Beamline 4-1 while the 2-year-burned samples were analyzed at Beamline 2-3 at SSRL, as described below.

Data reduction was completed using standard procedures.^39^ Briefly, replicate scans (4–5) were averaged to improve the signal-to-noise ratio and dead-time corrected when acquired using the Ge detector. No X-ray-induced changes were observed between the replicate scans. X-ray absorption spectra were averaged, background subtracted and normalized to an edge step of 1.0 using Sixpack (V.156).^40^ The pre-edge region was fit with a linear function from 7020 to 7070 eV for Fe and 6350 to 6500 eV for Mn, while the post-edge region was fit with a quadratic function from 7381 to 7070 eV for Fe and 6936 to 6500 eV for Mn. Values of E0 of 7125 eV for Fe and 6550 for Mn were used for data reduction.^40^ Principal component analysis (PCA) and target transform analysis were then performed in Sixpack (V.156).^40^ to identify a set of reference spectra for linear combination fitting (LCF) of the Fe and Mn K-edge XANES spectra acquired from the various samples (Tables S3 and S4). Reference spectra for use in LCF were selected using principal component analysis (PCA) and target transform analysis, where the resulting ‘spoil’ value obtained for each target transform was used to exclude phases that could not be reconstructed using the principal components. Additional candidate standards were also tested during LCF analysis but did not improve the R-factor or yield unrealistic component proportions. All LCF analysis was performed in Athena.^41^

#### 2.3.5 Micro-XRF Mapping and Micro-XANES Analyses

The elemental distribution and microscale speciation of Fe and Mn in ash samples were analyzed using micro-XRF and micro-XANES analyses at Beamline 2-3 at SSRL using a Si(111) monochromator. Monochromator energies for Fe and Mn were calibrated in the same way as those for the bulk Fe XANES analysis. Samples were embedded in epoxy (Epotek 301), mounted on a quartz slide, and polished to a thickness of 30 μm (Grindstone Laboratory; Portland, OR). These thin sections were imaged with a Vortex silicon drift detector using a 5 μm diameter beam. The micro-XRF maps were collected under ambient conditions at an incident energy of 7200 eV with a 25 ms dwell time and a 10 μm step size, which provided elemental distributions for Mn, Fe, Al, Si, and Ca. Subsequently, point micro-XANES spectra at the Fe and Mn K-edges (4–6 per sample, per edge) were collected from locations with distinct elemental associations or particle morphologies. Fe and Mn K-edge bulk XANES spectra of the 2-year-burned samples were also collected at Beamline 2-3 instead of Beamline 4-1 due to beam time constraints. For these measurements, the soil was spread on Kapton tape, and Fe and Mn K-edge XANES spectra were measured at room temperature with a 1 mm × 1 mm beam. Data reduction was completed using the procedure described in Section 2.3.1, and the results of principal component and target transform analysis are available in Tables S5 and S6.

#### 2.3.6 Scanning Transmission X-ray Microscropy (STXM)

Scanning transmission X-ray microscopy (STXM) was used to investigate microscale variations and elemental associations of C (K-edge), Ca (L-edge), Fe (L-edge), and Mn (L-edge) in chaparral and fir samples, with sample preparation, measurement conditions, and image analysis described in detail in Note S3.

### 2.4 Metal Mobilization Experiments

We conducted batch kinetic experiments to assess the mobility of Fe and Mn from a subset of samples in the absence and presence of pyoverdine (50 μM, added after overnight equilibration). All metal mobilization experiments were performed in duplicate. All experiments were conducted using a 1 g L^–1^ suspension density under ambient atmospheric conditions and constant temperature (20 ± 1 °C). An ionic strength equivalent to 0.03 M NaCl was used for all treatments. Batch reactors (glass beaker, 100 mL) were wrapped in aluminum foil to prevent any photoinduced redox reactions. The resulting suspensions were agitated continuously for 48 h using a magnetic stir bar and a Teflon-coated stir bar. Sample aliquots were collected and passed through a 0.22 μm poly­(ether sulfone) (PES) filter prior to analysis by ICP-MS. Additional information is provided in Note S4.

## 3 Results and Discussion

### 3.1 Elemental Composition and Origin of Fe and Mn Species in Wildfire Ash

The compositions of all samples used in this study are summarized in Figure 1. The concentrations of Al, Ti, and Fe were similar in the ash and 2-year-unburned surface soils and less abundant by 1–2 orders of magnitude in the vegetation samples (Figure 1a, Table S7). While we did not determine the Si content for our vegetation samples, we expect that Si in the vegetation samples will be small compared to the soil and ash samples. Published values indicate less than 2 wt % for needles/leaves and one to 2 orders of magnitude lower for branches.^42^ Taken together, these data indicate that Si, Al, Ti, and Fe in ash are derived primarily from soil minerals. This hypothesis is supported by correlation plots of Fe against Al, Ti, and Si, which show similar trends for the soil and ash samples (Figure 1b–d), although the Oak sample appears as an outlier with a higher Fe/Si and Fe/Ti ratio than the other ash samples. Instead, the ash samples were enriched in several biogenic elements (Figure 1a and Table S7), with about 5-fold higher concentrations of Ca and 3- to 4-fold higher concentrations of P, K, S, and Mn in the ash relative to the unburned soils. The lower enrichment of the ash in Mn compared to that in Ca suggests that Mn is derived from both biomass and minerals. However, soil minerals were not the main source of Mn in three of the four ash samples as indicated by correlation plots of Mn against Al, Si, and Ti (Figure 1e–g). Only the Pond sample falls within the 95% confidence envelope of the soil samples. This result can be rationalized by the low amount of biomass around the retention pond relative to the densely vegetated areas prefire from which the Fir, Oak, and Chaparral samples derived. The transfer of (micro)­nutrients from above-ground biomass and soil minerals into ash suggests a significant impact of wildfires on soil biogeochemistry. In particular, the weathering of Fe- and Mn-bearing species in ash could deliver a quantitatively significant pulse of these micronutrients to the underlying A horizon (top soil) in the early stages of soil and vegetation recovery. This effect, however, is transient as the composition of the 2-year-burned soils approaches that of the 2-year-unburned soils (Figure 1a and Table S7).

**Figure 1:**
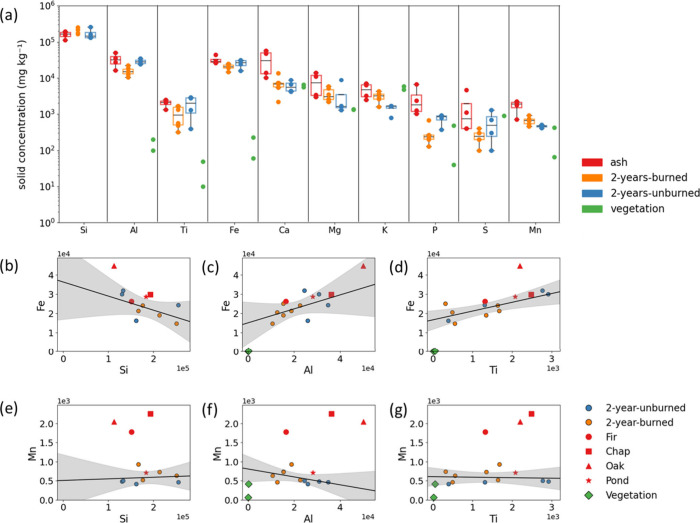
Total concentrations (a) of Si, Al, Ti, Fe, Ca, K, Mg, P, S, and Mn in ash (*n* = 4), 2-year-burned (*n* = 6), 2-year-unburned (*n* = 4), and vegetation samples (*n* = 2). The *y* axis is logarithmic, and data are summarized using box and whisker plots. Note that Si concentration values are not available for the vegetation samples. Scatter plots showing the relationships between Fe concentration and Si (b), Al (c), and Ti (d), and Mn concentration and Si (e), Al (f), and Ti (g) across different sample types except vegetation samples. Circles represent surface soils samples (2-year-unburned in blue and 2-year-burned in orange), and diamonds represent vegetation samples. Ash samples are shown as red markers (Fir: circle, Chaparral: square, Oak: triangle, Pond: star). The shaded regions in parts b–g represent 95% confidence intervals for linear regression fitted only to 2-year-unburned and 2-year-burned soils. Two elements, Si and Ti, were measured using portable X-ray fluorescence (pXRF), while all other elements were quantified using inductively coupled plasma atomic emission spectroscopy (ICP-AES) and mass spectrometry (ICP-MS) following aqua regia digestion of the samples.

### 3.2 Fe Speciation in Wildfire Ash and Surface Soils

We examined the average speciation of Fe in ash and surface soil samples using bulk Fe K-edge XANES spectroscopy. Figure 2a shows the first derivatives of all sample and reference Fe K-edge XANES spectra (Figure S2a), with the corresponding LCFs. Reference spectra for LCFs were selected based on PCA and target transform analysis using all sample spectra, which returned spoil values 2.1, 2.0, and 1.2 for hematite, two-line ferrihydrite, and maghemite, respectively (Table S3). Although goethite is a common Fe­(III) (oxyhydr)­oxide in soil, its high spoil value (3.4) and absence when included in LCFs indicate that goethite was not detectable in any of our samples. Based on LCF analysis, Fe in ash samples occurred as two-line ferrihydrite (17–48%), hematite (16–49%), and maghemite (19–43%), resulting in a component sum of 97–100% and R-factor values of less than 0.0008 (Figure 2b). Although ash from wildland and urban interface fires can contain magnetite [i.e., Fe^2+^(Fe^3+^)_2_O_4_],^31^ a partially reduced Fe phase, the relatively high spoil value for magnetite (2.4) and its absence when included in LCFs indicate the absence of magnetite in our samples. In contrast, the spectra for the 2-year-burned and 2-year-unburned samples were reconstructed using the spectra of two-line ferrihydrite (32–77%) and hematite (17–70%), resulting in a component sum of 96–100% and R-factor values of less than 0.01 (Figure 2b); no maghemite was required to fit the spectra from these samples.

**Figure 2:**
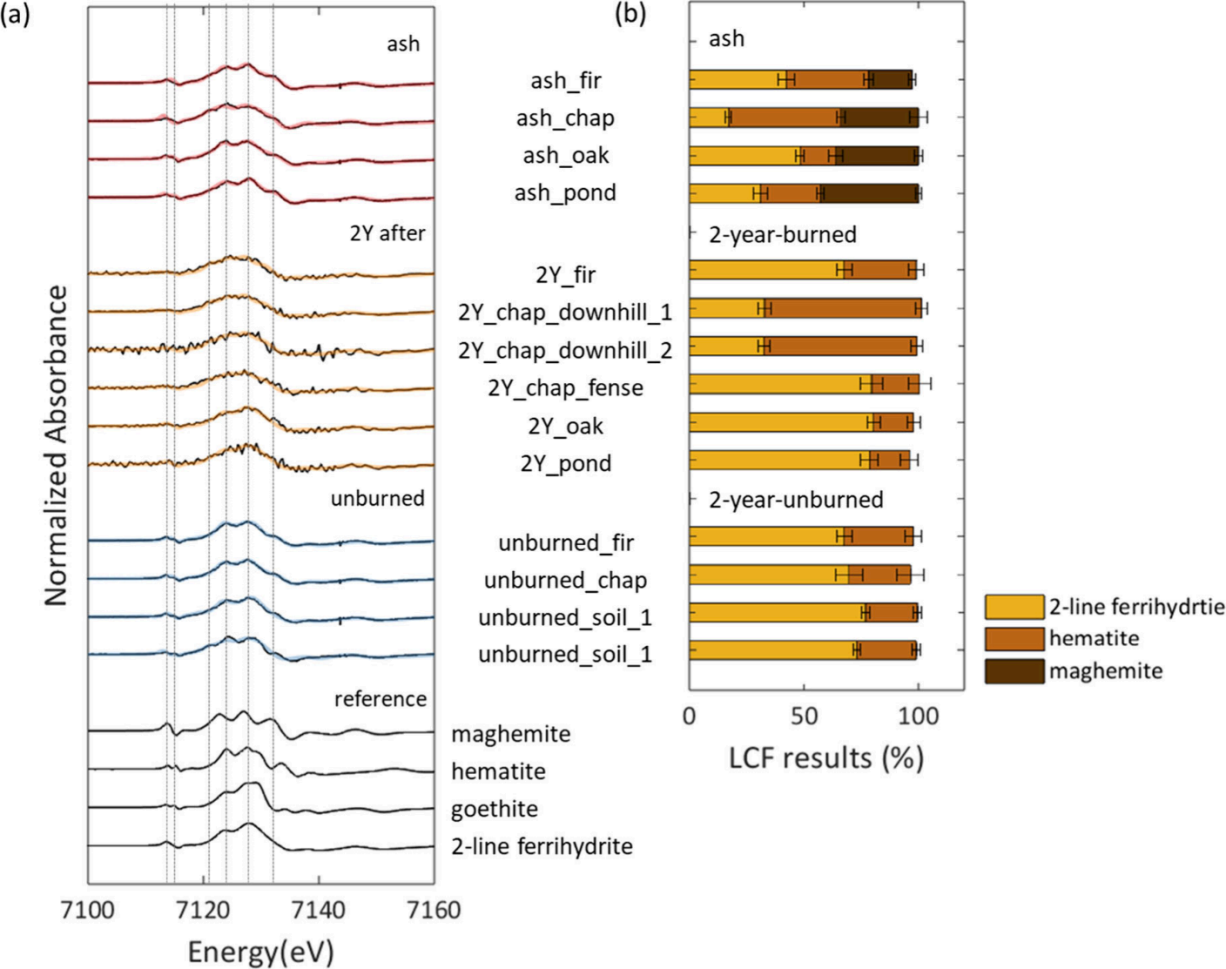
Iron speciation in wildfire-impacted and unburned soils. (a) First derivatives of bulk Fe K-edge XANES spectra (Figure S2a) for ash (*n* = 4), surface soil collected two years after the wildfire (2-year-burned, *n* = 6), and surface soil from unburned areas collected at the same time (2-year-unburned, *n* = 4). Black lines represent measured data and red, orange, and blue lines represent linear combination fits for ash, 2-year-burned, and 2-year-unburned, respectively. Reference spectra of Fe mineral standards are shown in thin black. Measured spectra for the 2-year-burned soils exhibit greater noise due to the use of microfocused XANES at Beamline 2-3 (SSRL), in contrast to bulk XANES analysis of other samples conducted at Beamline 4-1. (b) Quantitative Fe speciation results from linear combination fitting of XANES spectra, showing the relative proportions of two-line ferrihydrite, hematite, and maghemite. Error bars indicate the fitting uncertainties for each component.

In addition to the average Fe speciation, we analyzed the ash particles using micro-XRF and micro-XANES analysis. Generally, Fe was colocated with Al (i.e., yellow-green areas) but not Si, which occurred as distinct particles (Figure 3a,c,e,g). The colocation of Fe and Al may arise from the aggregation of particles with distinct elemental composition or the occurrence of Al-substituted Fe­(III) minerals.^43^ Like the bulk XANES spectra, the μ-XANES spectra collected from these samples were best described by ferrihydrite, hematite, and maghemite (Figure 3b,d,f,h; Table S5), with two notable differences. First, the LCFs of the micro-XANES spectra indicated a higher fraction of maghemite compared to the bulk XANES analysis (Figure 3b,d,f), which may indicate differences in the size of maghemite and hematite particles. Second, LCF analysis of the Pond sample required the inclusion of lepidocrocite (up to 20%), which may result from the lower burn severity, higher moisture content, or distinct prefire Fe speciation in this riparian zone relative to the other samples.

**Figure 3:**
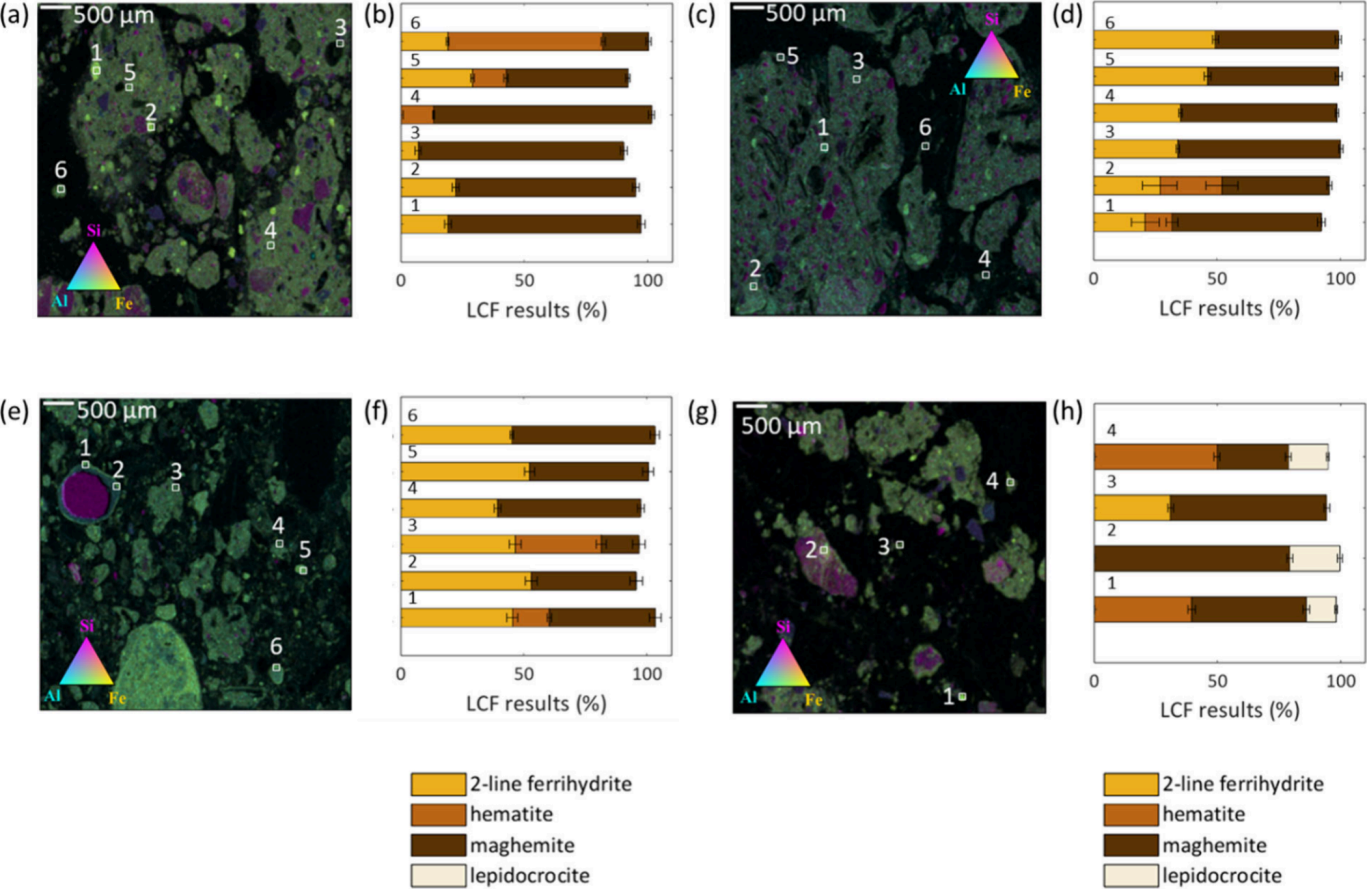
Tricolor maps and Fe speciation of wildfire ash samples. (a, c, e, g) Elemental distribution maps showing the colocation of Fe (yellow), Al (cyan), and Si (magenta) in ash particles from different vegetation types: (a) Fir, (c) Chaparral, (e) Oak, and (g) Pond. (b, d, f, h) Corresponding Fe mineral speciation results from linear combination fitting (LCF) of Fe K-edge XANES spectra at selected points (indicated on the maps and detailed in Figure S3). Bars represent the fractional contribution of four Fe phases: two-line ferrihydrite, hematite, maghemite, and lepidocrocite. Error bars indicate fitting uncertainties for each component. The fitting error for each Fe component is plotted as an error bar.

The occurrence of maghemite and hematite in wildfire ash is consistent with laboratory experiments. While synthetic ferrihydrite transforms to hematite when heated at 200–680 °C under oxidizing conditions,^44,45^ ferrihydrite in soil transformed into maghemite instead of hematite.^46^ The presence of maghemite in our samples thus may result from the heating of Fe-bearing minerals in close association with organic matter. Pyrogenic maghemite, however, can further transform to hematite at temperatures of 360–600 °C.^44–46^ Higher burn severities would thus favor the transformation of pyrogenic maghemite to hematite. In the ash samples, both maghemite and hematite appear to be pyrogenic. The absence of maghemite in all 2-year-burned samples indicates that maghemite is a transient phase, which undergoes lateral or vertical translocation by wind or precipitation and/or chemical transformation. The latter hypothesis is corroborated by the similar total Fe content but higher fraction of hematite in the downhill 2-year-burned surface soils (up to 70%) compared to the uphill 2-year-burned sample (<31% hematite). Overall, the data presented here suggests that wildfire replaces a moderately soluble and poorly crystalline Fe­(III) phase [2-line ferrihydrite (log *K*_sp_ = 3.54)] with less soluble phases [maghemite (log *K*_sp_ = 1.59) and hematite (log *K*_sp_ = 0.09)].^47,48^ The transformation of Fe minerals into more thermodynamically stable forms may alter Fe availability for plants and microbes in soil, particularly under the high pH conditions that result from ash-driven alkalinization.^8,20^

### 3.3 Mn Speciation in Wildfire Ash and Surface Soils

The first derivatives of the bulk Mn K-edge XANES spectra (Figure S2b) collected from the ash, 2-year-burned, and 2-year-unburned samples and corresponding LCFs are shown in Figure 4a. Reference spectra for LCF analyses were selected based on PCA using all sample spectra and subsequent target transform analysis (Table S4), which returned spoil values of 0.1, 2.1, 3.0, and 3.2 for δ-MnO_2_, hausmannite, Mn­(III)-acetate (Mn­(III)-organic surrogate), and MnSO_4_ (Mn­(II) surrogate), respectively. Linear combination fits of the Mn K-edge spectra revealed three dominant species in the ash samples: Mn­(III)-organic (17–34%), hausmannite (42–60%), and Mn­(II) (23–31%), resulting in a component sum of 98–100% and R-factor values of less than 0.001 (Figure 4b). In contrast, the 2-year-burned surface soils showed a higher contribution of Mn­(III)-organic species (up to 64%), a lower fraction of MnSO_4_ (Mn­(II), 2–19%) and no hausmannite (Figure 4b). These samples also showed characteristic features of δ-MnO_2_ (32–65%), an analogue for layer-type Mn oxides common in soil (Figure 4a,b). Similarly, two of the 2-year-unburned surface soil samples were composed of δ-MnO_2_ (>99.1%) and Mn­(II) species (<10%), with no apparent contribution from hausmannite. The other two 2-year-unburned surface soil samples, which had some litter fragments, contained 44–56% Mn­(III)-organic species and either Mn­(II) (21–44%) or hausmannite (35%). Although hausmannite could account for some of the spectral patterns attributed to Mn­(III)-organic and Mn­(II), the consistent fitting of Mn­(III)-organic across all ash and litter-containing soil samples and the absence of δ-MnO_2_ in these samples support the interpretation that the burning of litter and/or vegetation produces mixed-valent Mn­(II,III) phases.

**Figure 4:**
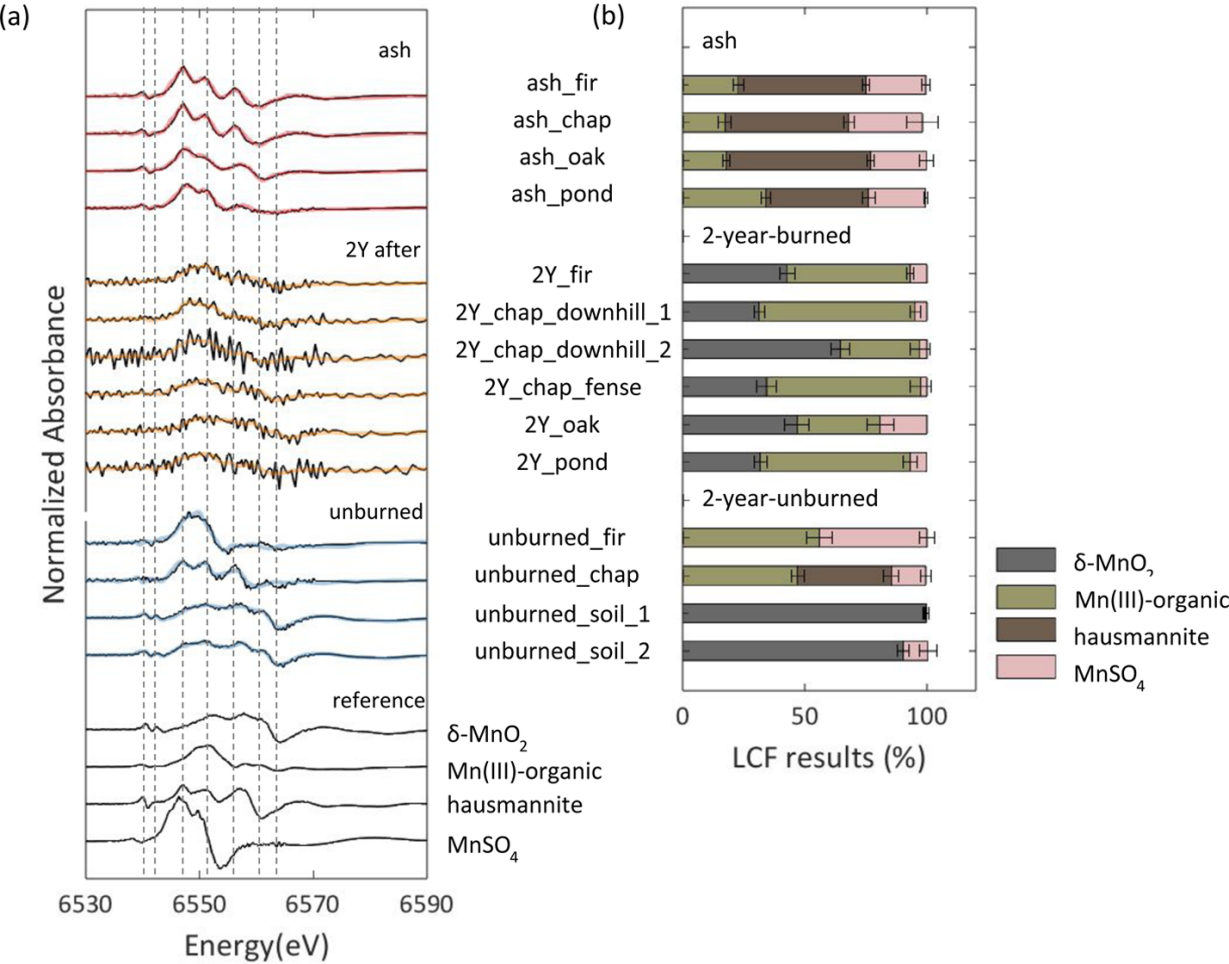
Manganese speciation in wildfire ash and soil samples. (a) First derivatives of bulk Mn K-edge XANES spectra (Figure S2b) from ash (*n* = 4), surface soil collected two years after the wildfire (2-year-burned, *n* = 6), and unburned surface soil collected at the same time (2-year-unburned, *n* = 4). Black lines represent measured data and red, orange, and blue lines represent linear combination fits for ash, 2-year-burned, and 2-year-unburned, respectively. Measured spectra for the 2-year-burned soils exhibit greater noise due to the use of microfocused XANES at Beamline 2-3 (SSRL), in contrast to bulk XANES analysis of other samples conducted at Beamline 4-1. Thin black lines show reference spectra for δ-MnO_2_, Mn­(III)-organic complexes (Mn­(III)-acetate), hausmannite, and MnSO_4_. (b) Results of linear combination fitting, showing the relative contributions of four Mn species: δ-MnO_2_, Mn­(III)-organic, hausmannite, and MnSO_4_. Error bars indicate fitting uncertainties for each component.

The distribution and speciation of Mn in the ash particles were also examined using μ-XRF and μ-XANES, respectively (Figure 5). Tricolor maps showed that Mn occurred either as single particles (appearing as magenta) or admixed with Fe (appearing as orange), which may reflect the organic and mineral sources of Mn in the ash, respectively. No colocation between Mn and Ca was observed (appearing purple), despite their shared source (e.g., litter and vegetation), suggesting that burning leads to the formation of discrete phases, which condense at different temperatures. Manganese K-edge micro-XANES spectra revealed the same Mn phases identified by bulk Mn K-edge spectroscopy, but required the inclusion of the Mn­(III) oxide, bixbyite (Mn_2_O_3_(s), four out of 26 locations), and δ-MnO_2_ (six out of 26 locations) (Figure 5b,d,f,h, Table S6). Evidently, bixbyite and δ-MnO_2_ are minor phases present below their detection limit (>5% mass) for bulk XANES analysis, but appear as discrete particles detectable by μ-XANES. Across all samples, hausmannite (up to 74%), Mn­(III)-organic (up to 48%), and MnSO_4_ (up to 45%) were commonly detected at the same location. In contrast, δ-MnO_2_ and bixbyite were spatially localized and consistently colocated with Fe (positions 1, 2, 3, and 6 in Figure 5a and positions 2, 3, and 6 in Figure 5c). Compared to the high burn severity samples, the Pond sample exhibited a greater variety of Mn phases, including δ-MnO_2_, bixbyite, Mn­(III)-organic, hausmannite, and Mn­(II), suggesting that the moderate burn severity and increased moisture at this location preserve native Mn phases, while also giving rise to pyrogenic Mn phases.

**Figure 5:**
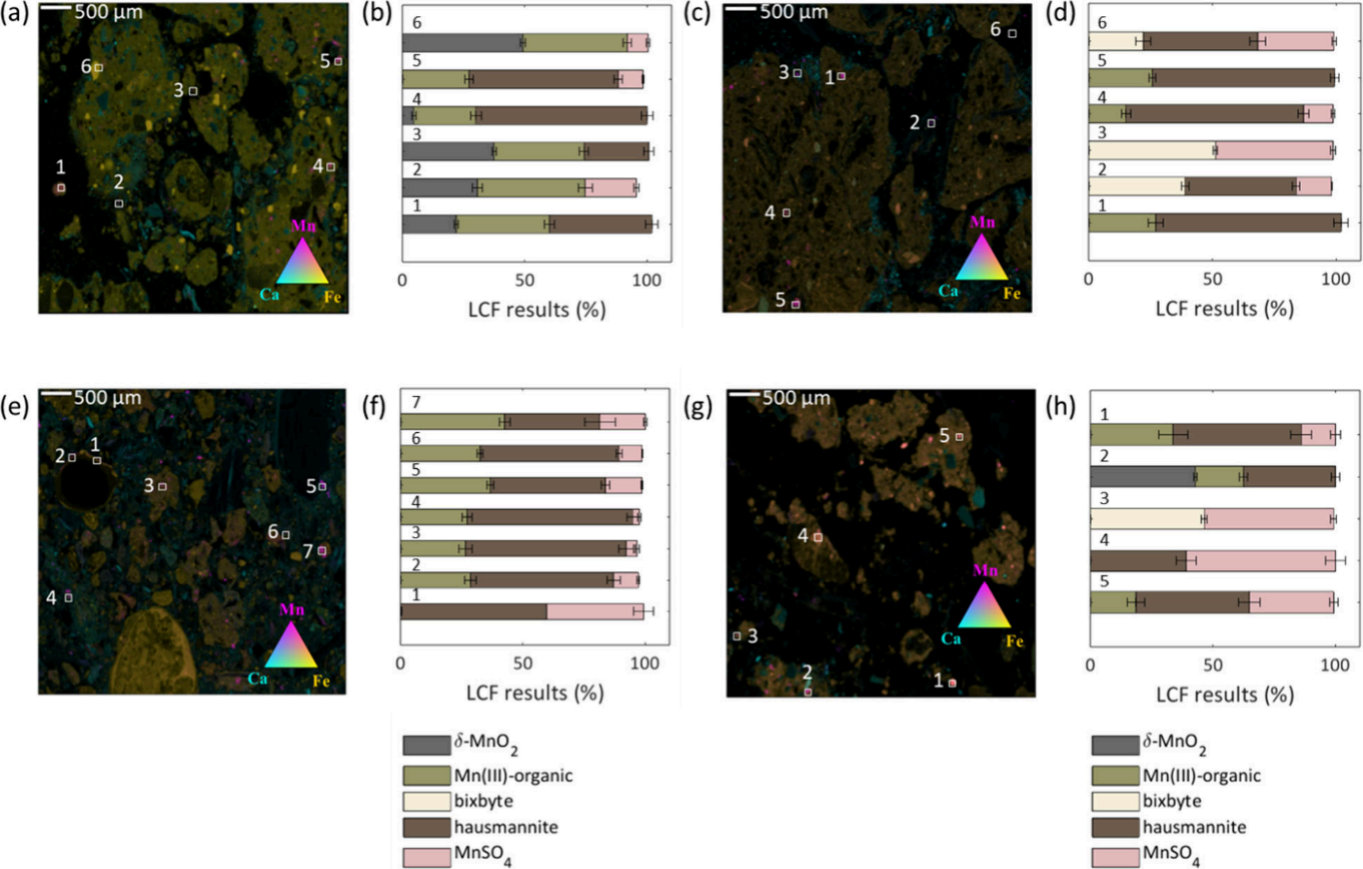
Tricolor maps and Mn speciation of wildfire ash samples. (a, c, e, g) Elemental distribution maps showing the colocation of Mn (magenta), Ca (cyan), and Fe (yellow) in ash particles from different vegetation types: (a) Fir, (c) Chaparral, (e) Oak, and (g) Pond. (b, d, f, h) Manganese speciation results based on linear combination fitting (LCF) of Mn K-edge XANES spectra collected from selected points indicated on the maps and detailed in Figure S4. LCF results show the fractional contributions of δ-MnO_2_, Mn­(III)-organic complexes, bixbyite, hausmannite, and Mn­(II). Error bars indicate the fitting uncertainties for each Mn species.

Wildfire transformed Mn in the vegetation, litter, and soil into more thermodynamically stable forms. In the ash, mixed-valent Mn phases such as hausmannite and bixbyite were dominant. Hausmannite and bixbyite are typically found in hydrothermal and metamorphic deposits,^49,50^ while Mn­(II) and Mn­(III)-organic complexes are often found in vegetation.^51^ The lower fraction of Mn­(III)-organic and the large fraction of hausmannite and bixbyite in the ash samples compared to the unburned samples can be explained by thermal transformation of Mn­(II) and Mn­(III)-organic species during the fire. Additionally, Cheraghi et al. reported that calcination of MnO(s) in the presence of Fe leads to its transformation to bixbyite at temperatures above 350 °C and hausmannite at temperatures above 550 °C.^52^ The formation of new Mn­(II,III) minerals during wildfire may alter Mn bioavailability, necessitating new Mn uptake strategies by plants and microbes for postfire regeneration.

### 3.4 Micronutrient Mobilization from Wildfire Ash

To assess the potential mobilization of Fe and Mn from the ash samples relative to 2-year-burned and unburned surface soils, we conducted batch dissolution experiments in the absence and presence of 50 μM PVD at a 1 g L^–1^ solids concentration (Figure 6 and Figure S5). These experiments were conducted at a pH value of 8.0 to match the pH of the ash samples, which, on average, was 1–3 pH-units higher than the 2-year-burned (on average 6.6) and unburned samples (on average 5.9), as shown in Figure S6. In the absence of PVD, we found up to 0.3–2.1, 1.5–2.3, and 1.4–6.9 μM mobilized Fe concentrations from the ash, 2-year-burned, and unburned samples, respectively. A similar trend was observed in separate experiments (1:2.5 solid concentration, Table S8), where Fe mobilization from the surface soils was 10-fold higher than from the ash samples. These Fe concentrations measured in solution (Figure S9) exceed the solubility of typical Fe­(III) oxyhydroxides of 10^–8^–10^–9.5^ M at pH 6.0–8.0. When normalized to total Fe content, Fe mobilization from the ash samples ranged from 0.01–0.04 mol kg^–1^ compared to 0.05–0.09 mol kg^–1^ for 2-year-burned and 0.07–0.1 mol kg^–1^ for unburned soils (Figure 6). These data also indicate lower mobilization of Fe from the ash than soil samples, which is consistent with the greater fraction of less soluble Fe­(III) oxides in the ash samples (Figure 6). Given the low solubility of Fe­(III) in the absence of high Fe affinity ligand^47^ and absence of more soluble Fe­(II) in our samples (see Section 3.2), the mobilized Fe likely comprises labile Fe­(III)-OM complexes or colloids not retained during separation of the solution phase (i.e., <0.22 μm filter). The occurrence of less soluble Fe­(III) phases (Figures 2 and 3) and loss of Fe-OM associations, as indicated by our STXM analysis (Figure S7) in the ash samples, may explain the lower mobilization of Fe from the ash samples relative to the soil samples.

**Figure 6:**
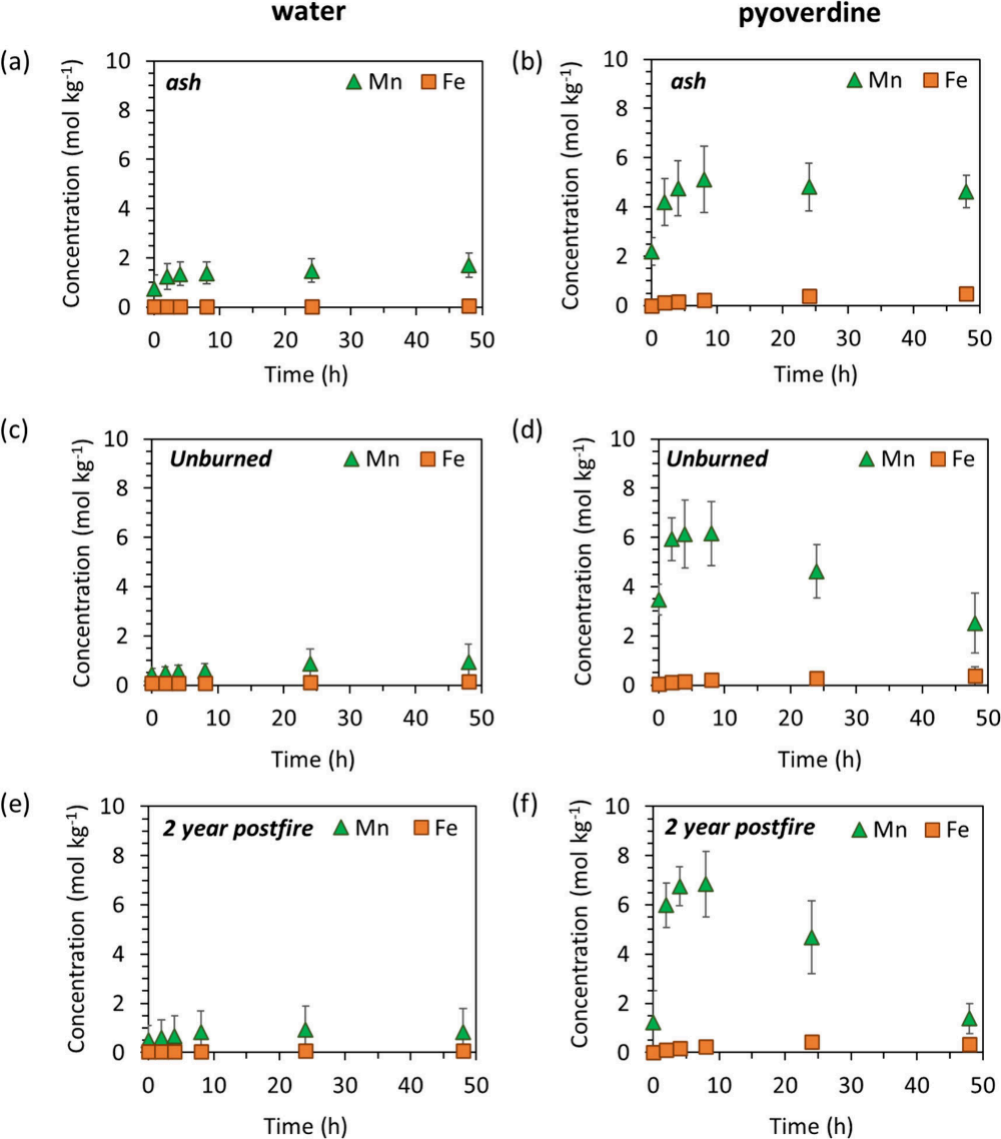
Total solid concentration normalized Fe and Mn mobilization from (a, b) ash (*n* = 4), (c, d) surface soil that was not burned during the wildfire and collected two years after (2-year-unburned, *n* = 2, unburned Fir and Chaparral), and (e, f) surface soil two years after the wildfire (2-year-burned, *n* = 3, 2Y-Fir, 2Y-Chaparal-fence, 2Y-Pond) at pH 8.0 under oxic conditions (1 g L^–1^ suspension density). The error bars represent the differences between the samples.

The addition of 50 μM PVD to the ash and soil suspensions increased the mobilization of Fe by up to a factor of 12, 4.0, and 1.6 for ash, 2-year-burned, and unburned samples (Figure S6). Normalized Fe release reached up to 0.6 mol kg^–1^ of total Fe in the ash suspensions—a significantly higher mobilization extent than observed for the soils (0.04–0.3 mol kg^–1^ for burned and 0.002–0.3 mol kg^–1^ for 2-year-burned)—despite similar bulk Fe contents (Figure 6 and Figure 1). The higher mobilization of Fe from the ash samples upon addition of PVD is notable not only because all sample types had similar solid-phase Fe concentrations (Figure 1), but also because the ash samples contained large fractions of less soluble Fe phases (i.e., maghemite and hematite) compared to the unburned and 2-year-burned samples (Figure 2). The enhanced reactivity of PVD toward the Fe phases in the ash samples may be due to high reactive surface area^53–55^ of Fe-bearing minerals created during wildfire due to structural and morphological changes and the loss of aggregate structure^56,57^ as organic matter is combusted and mineral surfaces become exposed, as suggested by absence of Fe–C associations in our STXM images (Figure S7). Both mechanisms would lead to enhanced adsorption of PVD on mineral surfaces and enhanced Fe mobilization.^58,59^ Iron mobilization by PVD may be further enhanced by the synergistic interaction of the ligand and dissolved organic carbon with the Fe­(III) minerals.^60–62^ In fact, the water-extractable dissolved organic carbon was about 10 times greater from the ash samples than from the unburned surface soil samples, notwithstanding the similarity in the solid-phase organic carbon content for both sample types (Figure S8a,b).

For Mn, the maximum mobilized concentrations in the absence of PVD were 1.6–3.8, 0.1–1.5, and 0.2–0.6 μM from ash, 2-year-burned, and unburned samples, respectively. When normalized to total Mn content, the ash released 0.7–1.7 mol kg^–1^, which was greater than the observed 0.5–0.9 and 0.4–1.0 mol kg^–1^ from 2-year-burned and unburned soils, respectively (Figure 6). The higher mobilization of Mn from the ash, even on a normalized basis, is consistent with the predominance of more soluble Mn­(II) and Mn­(III) species (e.g., hausmannite and bixbyite) relative to the Mn­(III,IV) species in soils. Similarly, experiments using a higher solids concentration showed similar results, with 4.5 times more Mn mobilized from the ash samples than surface soil samples (Table S8). Upon addition of 50 μM PVD, Mn mobilization increased by a factor of up to 2.8, 1.7, and 3.7 for the ash, 2-year-burned, and unburned samples, respectively, with the highest amount of Mn mobilized from the ash samples (up to 8.1 μM). When normalized to total Mn content, the ash released 2.1–5.1 mol kg^–1^, which was comparable with the 1.2–6.8 and 2.5–6.2 mol kg^–1^ mobilized from 2-year-burned and unburned soils, respectively (Figure 6). This indicates that the greater absolute Mn concentrations observed in the ash suspensions primarily reflect the higher total Mn content rather than enhanced Mn solubility. While the ash samples contained relatively soluble Mn­(III)-organic compounds and Mn­(II) species,^62,63^ the dominant Mn phases in ash were more stable Mn­(II,III) oxide minerals such as bixbyite (log *K*_sp_ = −1.92) and hausmannite (log *K*_sp_ = −1.73),^63^ which typically exhibit low solubility. The high affinity of PVD for Mn­(III) and its ability to act as both reducing agent and ligand toward Mn­(III) and Mn­(II,III) oxide minerals,^32,64^ enhanced Mn mobilization from the ash and soil samples.

For both Fe and Mn, the extent of ligand-enhanced metal mobilization from the ash was higher than from either 2-year-burned or unburned soils. To assess whether the ash can contribute a significant pool of micronutrients to the soil, we compared the active metal concentration in a 10 cm thick ash layer to the 5 cm profile that we sampled, following eq 1 (Table S9). The mobilized metal concentrations were based on the average of the maximum mobilized concentrations across the different sample types.

1 $$\begin{array}{l} active\;metal\;concentration\;\left[\frac{\mu mol}{cm^{2}}\right]\;=\\ mobilized\;metal\;\left[\frac{\mu mol}{L}\right]\times \frac{1}{solid\;concentration}\;\left[\frac{L}{g}\right]\times thickness\;\left[cm\right]\times density\;\left[\frac{g}{cm^{3}}\right] \end{array}$$

The active Fe concentration for ash samples ranges from 60 to 90 μmol cm^–2^ for ash densities of 0.4 to 0.6 g cm^–3^,^65,66^ respectively, which on average is 1.5 times higher than the active Fe concentrations of 39 and 58.5 μmol cm^–2^ that would be observed in soil with densities of 1.2 and 1.8 g cm^–3^, respectively.^67^ For Mn, the active concentrations from ash were, on average, 2.1 times higher than those in the soil. These estimates indicate that ash deposition, especially when leading to a thick ash layer, can increase the pool of Fe and Mn that can be readily mobilized in the soil in the presence of organic ligands.

### 3.5 Postfire Micronutrient Biogeochemistry

Wildfire can shift soil biogeochemical cycles by changing the composition and reactivity of soil components.^68^ For instance, fire changes the composition of soil organic carbon, leading to the formation of carbonate minerals and pyrogenic organic carbon residues, which alter soil pH and can serve as electron donor or acceptor compounds for microbial metabolism, respectively.^68^ Although Fe and Mn are critical in numerous soil processes,^25,26^ and laboratory heating experiments suggest that wildfire will transform Fe and Mn minerals in soil,^29,30^ limited research has examined their speciation and reactivity in fire-impacted soils.

Through the use of synchrotron-based methods, this study documented distinct Fe and Mn speciation in ash particles relative to that in soil. Our Fe K-edge XANES analysis showed that metastable ferrihydrite transforms into more stable phases like maghemite and hematite. For Mn, Mn K-edge XANES analysis showed that wildfire can lead to both oxidation [i.e., Mn­(II) to Mn­(III)] and reduction [i.e., Mn­(IV) to Mn­(III)] of Mn, resulting in large fractions of new Mn­(II,III) oxide phases like hausmannite. The generation of Fe and Mn phases with greater stability was accompanied by the loss of mineral-organic associations and increase in localized pH due to the formation of carbonate minerals and metal oxides. Given these properties, wildfire ash has the potential to shift postfire Fe and Mn cycling while impacting soil processes like nutrient cycling, organic matter turnover, and trace metal fate.

Despite the increased stability of Fe and Mn phases in the ash, we found that pyoverdine enhanced the mobilization of Fe and Mn from the ash to a greater extent than from the unburned surface soils. This result has significant implications for postfire ecosystem recovery, where plants and microbes can increase nutrient availability by exuding high-affinity ligands to access poorly accessible micronutrients.^69,70^ Moreover, previous studies have shown that wildfires generate pyrogenic organic carbon with increased aromaticity^71,72^ and greater electron-donating capacity^13,73^ compared to soil organic carbon. Our samples showed an order of magnitude greater lability of organic carbon from the ash samples than the surface soil samples, reflecting changes both in the chemical nature of the organic matter and loss of organo-mineral associations.^8,23^ This suggests that pyrogenic organic carbon is readily available and may enhance the reactivity of pyrogenic minerals in the presence of high affinity ligands such as siderophores,^61,74^ thereby enhancing the bioavailability of Fe and Mn. This process therefore may enhance early stage ecosystem recovery when conventional nutrient pools are disrupted.

Although the ash samples, collected shortly after the fire, showed distinct Fe and Mn speciation and reactivity, soils collected two years postfire exhibited Fe and Mn characteristics comparable to those of unburned soils. This loss of the ash layer from the land surface may be driven by erosion, translocation, and enhanced chemical weathering. While all three mechanisms can contribute to the loss of pyrogenic minerals, our work suggests that enhanced reactivity of Fe and Mn phases is driven by changes in mineralogy and aggregate structure that are driven by disruption in mineral–OM associations. Future work could focus on quantifying whether ash particles weather in place or are translocated to deeper layers and undergo weathering as they move through the soil profile. Additionally, understanding how the prevailing mechanism varies with precipitation regime will provide important insights into how pyrogenic residues may influence Fe and Mn bioavailability in the subsoil. This study provides an initial description of Fe and Mn speciation immediately and two years post fire, establishing boundary conditions that can guide future research on postfire biogeochemical cycles. Our findings highlight the dynamic and transient nature of postfire micronutrients (e.g., Fe and Mn) and provide a foundation for additional research that captures the effect of other variables across fire-impacted ecosystems. Building on this work, future research should employ larger sample sets where local vegetation type and soil burn severity are systematically characterized and incorporate time-series sampling to track changes from immediately after fire through multiyear recovery. Such efforts would also benefit from depth-resolved sampling to determine the extent to which fire alters soil structure, mineralogy, and organic matter composition as well as transect sampling across different vegetation types and landscape positions to better capture spatial heterogeneity. Finally, our data suggest that pyrogenic organic carbon may catalyze the dissolution of redox-active minerals generated during wildfire. Understanding this process will require a combination of laboratory dissolution experiments, time-series field sampling, and molecular-scale characterization of carbon and metal species to determine how pyrogenic organic matter alters mineral stability and postfire nutrient dynamics.

## Supplementary Material



## Notes

List of chemicals, sampling site description, sample types, pyoverdine preparation method, chemical analysis methods, description of batch dissolution experiments, total solid concentration of elements in samples, spoil values from target transform analysis of Fe and Mn, burned severity map, Pearson correlation heat map, pH, total organic and inorganic carbon concentration, dissolved organic and inorganic carbon concentration, tricolor map of C, Ca, and Fe, tricolor map of Fe, Si, and Ti, XANES spectra of Fe and Mn, mobilization kinetics of Fe and Mn, and active Fe and Mn concentration (PDF)
