# Supplementary material for: Wildfire Produces Transient Minerals: Speciation, Reactivity, and Fate of Iron and Manganese in Surface Soils Post Wildfire
Source: Environ Sci Technol. 2025 Dec 8;59(50):27342–53. doi: 10.1021/acs.est.5c07438 (PMC12750527; doi:10.1021/acs.est.5c07438)
Supplement: Supplementary file 1 [file es5c07438_si_001.pdf]

Supporting Information

Wildfire produces transient minerals: speciation, reactivity and fate of iron and manganese in surface soils post wildfire

Kyounglim Kang[1,2,3], Elizabeth M. Whelan[1], Sharon Bone[4,5], Mike C. Rowley[1,6,7], Matthew A. Marcus[2], Jasquelin Peña[1,6,*]

[1]Department of Civil and Environmental Engineering, University of California, Davis, California 95616, United States

[2]Advanced Light Source, Lawrence Berkeley National Laboratory (LBNL), Berkeley, CA 94720, USA

[3]University of Minnesota – Twin Cities, Department of Soil, Water, and Climate, 1991 Upper Buford Cir, St. Paul, MN 55108, United States

[4]Stanford Synchrotron Radiation Lightsource, SLAC National Accelerator Laboratory, Menlo Park, California 94025, United States.

[5]Institute of Bio- and Geosciences: Agrosphere, Forschungszentrum Jülich, Jülich 52428, Germany

[6]Earth and Environmental Sciences Area, Lawrence Berkeley National Laboratory, Berkeley, California 94720, United States

[7]Department of Geography, University of Zurich, Zurich 8057, Switzerland.

*Corresponding author, email: pena@ucdavis.edu

This PDF file includes:

Supplementary Methods: Notes S1-S5 (pages 2- 7)
Tables S1 to S9 (pages 8- 15)
Figures S1 to S9 (pages 16- 23)

Note S1. Pyoverdine preparation

Pyoverdine (PVD) was produced and purified according to Parker et al. (2004).[1] Briefly, *Pseudomonas putida* strain GB1 was grown an in iron-free defined medium containing 70 μM succinate and 70 μM L-proline as the carbon sources, 10 mM HEPES buffer set to pH 7, 0.4 mM $CaCl_3$, 0.25 mM $MgSO_4$, and trace metals ($CuSO_4 \cdot 5H_2O$, 273 μM $ZnSO_4 \cdot 7H_2O$, 84 μM $CoCl_2 \cdot 6H_2O$, and 53.7 μM $NaMoO_4 \cdot 2H_2O$, 5 mM $[NH_4]2SO_4$, 0.25 mM Na_2HPO_4, 0.15 mM KH_2PO_4). The bacteria were grown in the dark in a shaker incubator for a minimum of 72 h at 30°C. The pH was not controlled during growth. The bacteria were filtered with 0.22 μM disposable vacuum filter and the PVD-containing filtrate was freeze-dried and stored at -20°C. The supernatants were thawed, mixed with 0.3 g per of Chelex 100 resin (50-100 mesh, C77901-50G, Sigma) per 30 mL solution. The mixture was shaken intermittently (every 10 min) for 1 h at 4°C and filtered through 0.45 μM cellulose ester gridded filter paper (HAWG02500 Millipore) to remove divalent cations (e.g., magnesium and calcium) from the growth medium. The concentration of pyoverdine was determined by UV-vis spectrophotometry. The light green colored pyoverdine has an absorbance maximum at 400 nm (20,500 M^{-1} cm^{-1}) at pH 8.46.

Note S2. Climate and geographical information of field site

Local climate data were retrieved from the NOAA Climate Data Online (CDO) archive for the Santa Rosa, CA region. The climate in the study area is mesothermic with an average temperature that ranges between 7-34°C. The average annual precipitation prior to the fire was approximately 825 mm. In the two years following the Glass Fire (October 2020 to October 2022), cumulative precipitation was below average, with the region receiving 607 mm in 2020–2021 and 685 mm in 2021–2022, approximately 73-83% of the historical mean.[2]The sampled vegetation types include conifer, oak-forest, chaparral and riparian grasses adjacent to a retention pond. The soils underlying the vegetation have developed in sedimentary rocks of the Franciscan and Great Valley complexes, intermixed with younger, more recent volcanic deposits from the Sonoma Volcanic events.[3] Soils were sampled from locations near the steep terrain at the border between Pride Vineyard and Bothe-Napa Valley State Park and were shallow in nature (< 25-45 cm), containing an abundance of rock fragments (> 2 mm). The sampled soils were thus classified as Leptosols or Leptic Skeletic Cambisols (IUSS 2022).

Note S3. Scanning Transmission X-ray microscopy (STXM)

Scanning transmission X-ray microscopy was used to investigate microscale variation for C (K-edge), Ca (L-edge), Fe (L-edge) and Mn (L-edge) and their elemental association in the chaparral and fir samples. Samples were loaded onto transmission windows (Si_3N_4) using methods adapted from Chen et al. (2014).[4] Briefly 15-20 mg of sieved (2 mm) ash sample was vortexed with 1 mL Milli-Q H_2O (18.2 MΩ) for 10 s in an Eppendorf tube. 1 µL of sample suspension was then pipetted onto the Si_3N_4 windows, the edges of which were stuck over a sample holder with a mild adhesive.

Samples were measured at beamline (BL) 5.3.2.2, Advanced Light Source. Energy calibration was achieved by setting the $1s \rightarrow 3s\sigma_g$ C K-edge peak of CO_2 to 292.74 eV.[5] All image analysis was completed in STXM Image Reader using the methods outlined in Rowley et al. (2023).[6] Images obtained over a space of X and Y dimensions were taken above and below the K- or L-edge of different elements, background subtracted for I_0, and then subtracted from each other to give an elemental map for a specific element (C 295–280 eV; Ca 394.4–342 eV; Mn L-edge [2nd Harmonic] 318-321 eV; Fe 710–698 eV).

Note S4. Batch dissolution experiments

Batch dissolution experiments were conducted under controlled pH and background electrolyte concentrations. For ash samples, less than 0.01 M NaCl was added due to high electrical conductivity of the water extracts (2.4±1.1 mS cm^{-1}) of ash samples compared to the surface soil samples (0.27±0.17 mS cm^{-1}). For the PVD treatments, ash/soil suspensions were prepared using Mili-Q water given the high conductivity of the PVD stock solution (3.6 mS cm^{-1} equivalent to 0.03 M NaCl). The pH was maintained at 8.0 (ΔpH = ± 0.05), which was the average pH value of the ash extracts. by the addition of 10 mM HCl or NaOH, as required. The volumes of acid or base added for pH adjustment were recorded and accounted for when calculating the concentrations of all dissolved species. All suspensions were equilibrated overnight at 90 % of final suspension volume prior to ligand addition. A final volume of 50 mL and ligand concentration of 50 μM was reached after the addition of an aliquot from the ligand stock solution (between 800-900 μM). The initial time (t = 0) reported here corresponds to the end of overnight (12 h) equilibration period for the water-mobilization experiments or the moment of ligand addition for PVD treatments.

Dissolved Mn and Fe concentrations were determined by inductively coupled plasma mass spectrometry (ICP-MS, Agilent-7900). The instrument with a quartz spray chamber, a microMist concentric gas nebulizer, and nickel sampler and skimmer cones was operated using a 1.0 L min^{-1} flow rate of argon gas with insertion of helium gas at the reaction cell; the helium flow rate was set to 4.5 mL min^{-1} to diminish polyatomic interferences. The limit of quantification was 0.04 μg L^{-1} (0.67 nM) for Mn and 0.2 μg L^{-1} (4.4 nM) for Fe.

Table S1. List of chemicals.

Chemical	Formula	Supplier	CAS number	Purity
Sodium hydroxide solution	$NaOH$	Supelco	1310-73-2	1 M in H_2O
Hydrochloric acid solution	HCl	Supelco	7647-01-0	1 M in H_2O
Sodium chloride	$NaCl$	Sigma-Aldrich	7647-14-5	≥99.0%
Sodium citrate dihydrate	$Na_3C_6H_5O_7{\cdot}2H_2O$	Sigma-Aldrich	6132-04-3	≥99%
Cupric sulfate	$CuSO_4{\cdot}5H_2O$	Sigma	7758-99-8	≥98%
Zinc sulfate	$ZnSO_4{\cdot}7H_2O$	Sigma	231-793-3	≥99.0%
Cobalt chloride	$CoCl_2{\cdot}6H_2O$	Sigma-Aldrich	7791-13-1	98%
Sodium molybdenum oxide	$Na_2MoO_4{\cdot}2H_2O$	Sigma-Aldrich	10102-40-6	≥99%
Ammonium sulfate	$(NH_4)_2SO_4$	Sigma-Aldrich	7783-20-2	≥99.0%
Sodium Phosphate Dibasic Dihydrate	$Na_2HPO_4{\cdot}2H_2O$	Fischer chemical	10028-24-7	-
Potassium phosphate	KH_2PO_4	Sigma-Aldrich	7778-77-0	≥99.0%
Chelex 100 resin	$C_{10}H_{10}C_8H_8C_4H_7NO_4$	Millipore sigma	11139-85-8	-
HEPES	$C_8H_{18}N_2O_4S$	Sigma	7365-45-9	≥99.5%
Sodium succinate dibasic hexahydrate	$C_4H_4Na_2O_4{\cdot}6H_2O$	Sigma-Aldrich	6106-21-4	≥99%
L-Proline	$C_5H_9NO_2$	Sigma-Aldrich	147-85-3	≥99%
Calcium Chloride Dihydrate	$CaCl{\cdot}2H_2O$	Fischer chemical	10035-04-8	99.0-105.0%
Magnesium Sulfate	$MgSO_4$	ACROS Organics	7487-88-9	99%

Table S2. Description of sampling locations of ash samples and surface samples two years after within the Glass Fire burn perimeter, including pre-fire vegetation, burn severity, location and photographs taken.

Sample name	Fir	Chaparral	Oak	Pond
Previous vegetation	Fir	Chaparral	Shrub	Grass
Burn severity	Very high	Very high	Very high	Moderate
Burned area 2020	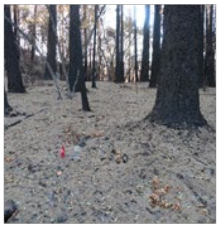	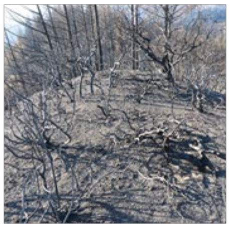	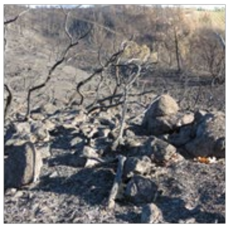	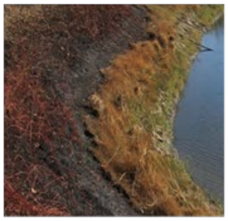
Burned area 2022	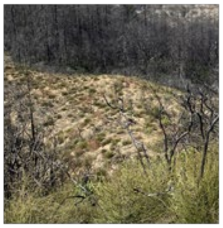	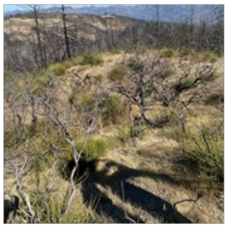	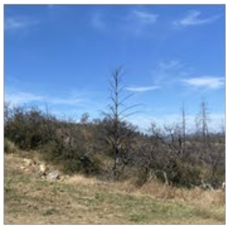	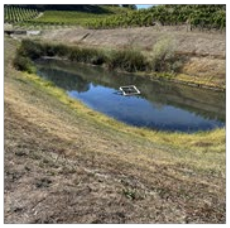
Location	38°31'40.0"N 122°33'58.0"W	38°31'45.0"N 122°34'08.0"W	38°31'34"N 122°33'49"W	38°31'40.0"N 122°33'52.0"W

Table S3. Description of sampling locations for ash, 2-year-unburned and 2-year burned samples.

types	Sample name	location
ash	Fir	38°31’40.0”N, 122°33’58.0”W
	Chaparral (chap)	38°31’45.0”N, 122°34’08.0”W
	Oak	38°31’34.0”N, 122°33’49.0”W
	Pond	38°31’40.0”N, 122°33’52.0”W
2-year-burned	2y_fir	38°31’40.0”N, 122°33’58.0”W
	2y_chap_downhill_1	38°31'38.0"N 122°33'60.0"W
	2y_chap_downhill_2	38°31'43.0"N 122°33'58.0"W
	2y_chap_fence	38°31'41.0"N 122°33'59.0"W
	2y_oak	38°31’34.0”N, 122°33’49.0”W
	2y_pond	38°31’40.0”N, 122°33’52.0”W
2-year-unburned	Unburned_fir	38°33’43.2”N, 122°34’47.5”W
	Unburned_chap	38°33’43.2”N, 122°34’35.1”W
	Unburned_soil_1	38°31'34.0"N, 122°34'2.0"W
	Unburned_soil_2	38°31'34.0"N 122°34'3.0"W

Table S3. The SPOIL values from the target transformation analysis of 3 principal components of the bulk Fe K-edge XANES spectral data using four Fe(III) reference spectra. (minimum energy: 7100, maximum energy: 7160 and k-weight: 3).

Standard	SPOIL value
Goethite	3.4
Hematite	2.1
Lepidocrocite	3.8
2-line ferrihydrite	2.0
Maghemite	1.2
Magnetite	2.4

Table S4. The SPOIL values from the target transformation analysis of 3 principal components of the bulk Mn K-edge XANES spectral data using four Mn reference spectra (minimum energy: 6530, maximum energy: 6580 and k-weight: 3)

Standard	Mn oxidation state	SPOIL value
δ-MnO_2	Mn(IV)	0.1
c-disordered birnessite	Mn(III) rich Mn(IV)	1.1
MnO_2 + glucose	Mn(III) rich Mn(IV)	1.2
Mn(III)-acetate	Mn(III)	3.0
Groutite	Mn(III)	3.7
Manganite	Mn(III)	9.7
bixbyite	Mn(III)	6.8
Hausmannite	Mn(II,III)	2.1
Rhodochrosite	Mn(II)	4.5
$MnSO_4$	Mn(II)	3.2

Table S5. The SPOIL values from the target transformation analysis of 3 principal components of the Fe K-edge micro-XANES spectral data using four Fe(III) reference spectra. (minimum energy: 7100, maximum energy: 7160 and k-weight: 3).

Standard	SPOIL value
Goethite	3.1
Hematite	1.8
Lepidocrocite	2.6
2-line ferrihydrite	1.8
Maghemite	2.4
Magnetite	5.0

Table S6. The SPOIL values from the target transformation analysis of 3 principal components of the bulk Mn K-edge micro-XANES spectral data using four Mn reference spectra (minimum energy: 6530, maximum energy: 6580 and k-weight: 3)

Standard	Mn oxidation state	SPOIL value
δ-MnO_2	Mn(IV)	2.3
c-disordered birnessite	Mn(III) rich Mn(IV)	2.2
MnO_2 + glucose	Mn(III) rich Mn(IV)	2.6
Mn(III)-acetate	Mn(III)	3.2
Groutite	Mn(III)	4.0
Manganite	Mn(III)	5.5
bixbyite	Mn(III)	4.2
Hausmannite	Mn(II,III)	3.8
Rhodochrosite	Mn(II)	4.8
$MnSO_4$	Mn(II)	3.3

Table S7. Total solid concentrations of major elements (Si, Al, Ca, K, Mg, Na, P, S and Ti) and elements of interest (Fe and Mn) in ash (n = 4), 2 years postfire surface soils (n = 6) and vegetation (n=2).

	OC	IC	Si	Al	Ca	K	Mg	Na	P	S	Ti	Fe	Mn
	%	%	ppm	ppm	ppm	ppm	ppm	ppm	ppm	ppm	ppm	ppm	ppm
**ash**													
chaparral	5.4	5.65	152000	16500	47500	6400	14100	350	2370	1100	1320	26300	1780
fir	2.57	1.77	194000	36200	14300	2500	3000	320	1270	400	2490	29800	2260
oak	3.24	5.41	113000	50100	57300	7100	11300	290	6600	4600	2200	44600	2050
pond	7.84	N/D	184000	28100	10400	3200	3400	280	1040	400	2080	28600	715
**2-year-burned**													
Chaparral fence	7.76	2.31	-	12600	5400	3600	5200	530	130	200	490	20500	462
chaparral downhill	5.7	2.24	-	15400	6700	3600	5900	600	260	100	320	25100	741
chaparral downhill_1	3.11	0.59	252000	10600	2200	4300	3500	350	200	N/D	560	14800	637
fir surface	6.6	2.22	214000	15300	13600	2800	2200	830	680	200	1350	19100	734
fir surface_1	5.48	1.13	167000	18900	7300	1600	2500	510	220	300	1680	21300	934
pond surface	7.45	2.29	177000	22600	6800	2600	2600	600	270	300	1640	24100	521
**2-year-unburned**													
unburned soil_1	7.88	5.56	131000	30900	4400	1700	1600	110	930	400	2920	30000	480
unburned soil_2	8.7	5.09	133000	24500	8900	1700	1700	90	800	300	2770	31900	505
Chaparral area	1.94	0.85	256000	34800	4200	1700	8900	150	370	100	1310	24300	461
Fir area	5.09	4.24	162000	25900	6700	800	1300	100	910	1300	390	16200	413
**Vegetation**													
chaparral	-	-	-	100	6500	4800	1350	1120	40	700	10	61	65.5
Fir	-	-	-	200	5600	5800	1370	1220	480	900	50	230	421

Table S8. Water extracted Fe and Mn concentration obtained from water extract (1:2.5, W/W) of ash (n = 4), 2 years postfire surface soils (n = 4) and vegetation (n=2).

	Fe<br>μM	Mn<br>μM
**ash**		
chaparral	N/D	9.4 (± 0.2)
fir	N/D	47.3 (± 1.0)
oak	N/D	13.0 (± 0.1)
pond	1.2	41.9 (± 0.4)
**2-year-burned**		
chaparral downhill	11.8 (± 5.8)	9.5 (± 1.8)
fir surface	2.4 (± 0.1)	2.1 (± 0.1)
fir surface_1	6.7 (± 3.9)	5.3 (± 0.0)
pond surface	10.8 (± 0.2)	30.7 (± 1.1)
**2-year-unburned**		
unburned soil_1	28.5 (± 18.7)	5.6 (± 0.4)
unburned soil_2	9.3 (±0.9)	5.5 (±0.0)
Chaparral area	5.1 (± 1.4)	1.9 (± 0.4)
Fir area	11.0	0.5

Table S9. Active Fe and Mn concentration calculated by Eq 1.

	mobilized conc. umol L^{-1}	solid conc. g L^{-1}	thickness cm	density g cm^{-3}	active conc. umol cm^{-2}	average
Fe ash	15.00	1.00	10.00	0.40	60.00	75.00
	15.00	1.00	10.00	0.60	90.00	
Fe unburned	6.50	1.00	5.00	1.20	39.00	48.75
	6.50	1.00	5.00	1.80	58.50	
Mn ash	8.10	1.00	10.00	0.40	32.40	40.50
	8.10	1.00	10.00	0.60	48.60	
Mn unburned	2.60	1.00	5.00	1.20	15.60	19.50
	2.60	1.00	5.00	1.80	23.40	

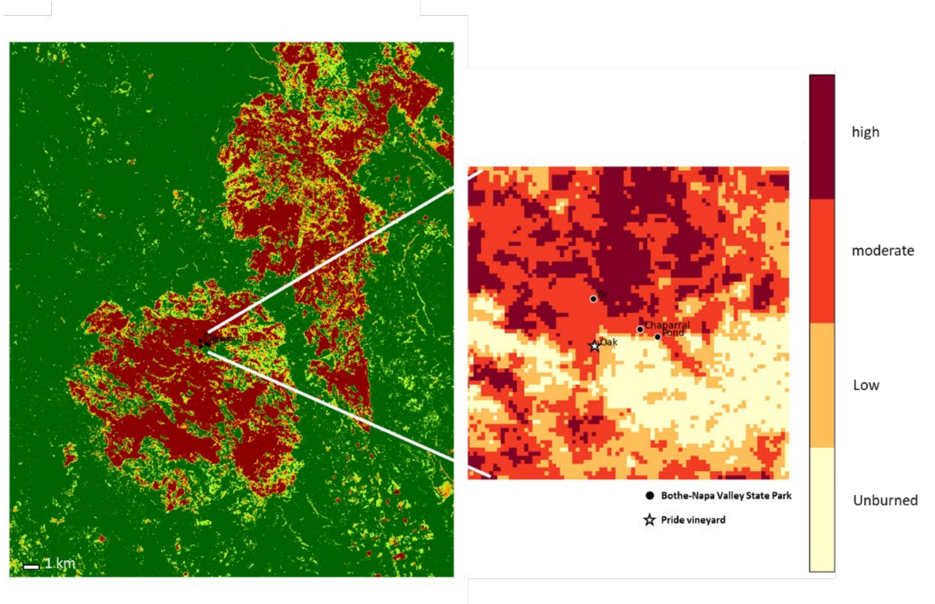

Sample name	Burn severity
Chaparral	moderate
Fir	moderate
Oak	moderate
Pond	low

Figure S1. The burn severity of Glass Fire ash sampling area.[7]

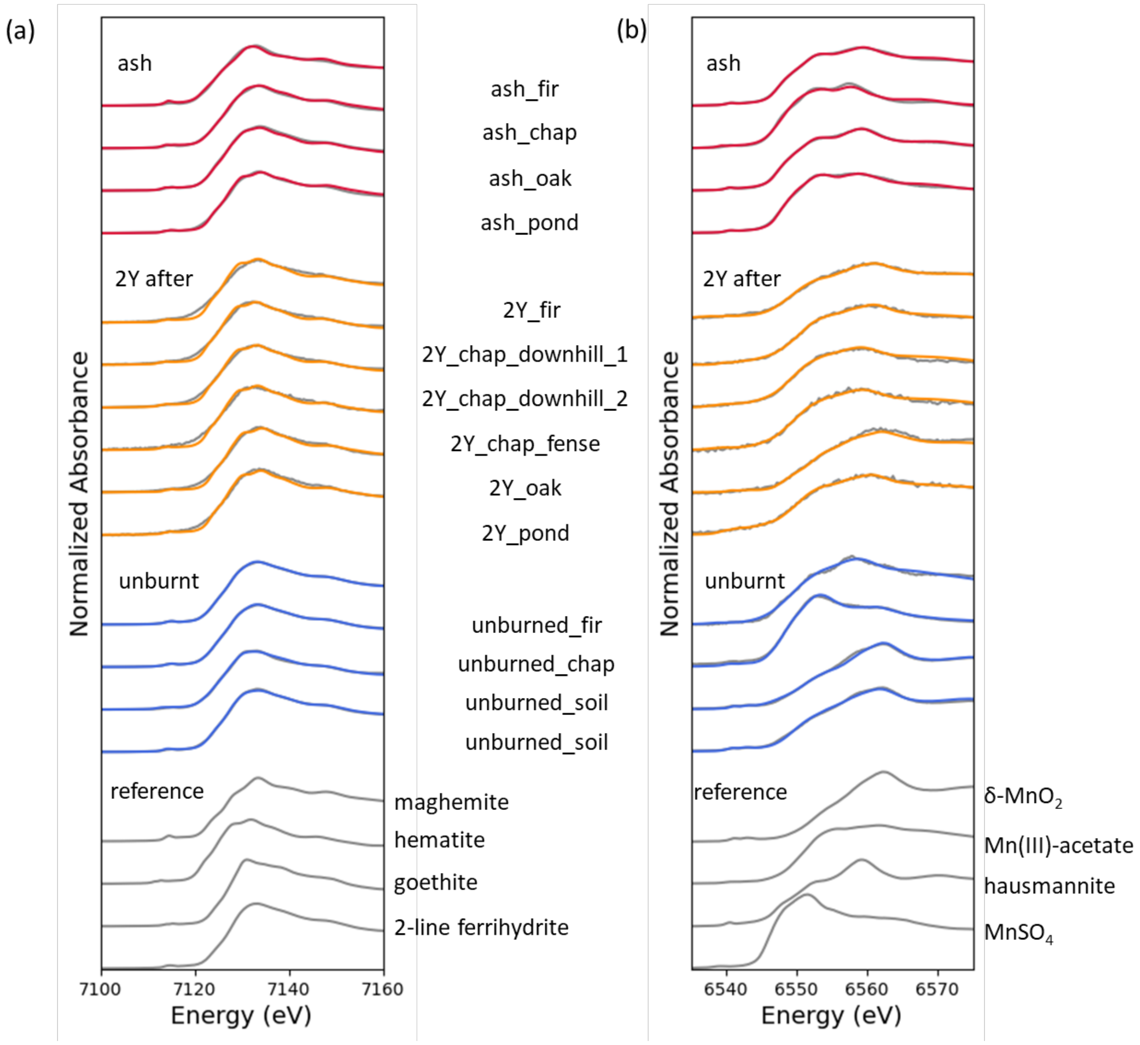

Figure S2. Iron (a) and manganese (b) K-edge XANES spectra of ash (n=4), surface soil 2-years after the wildfire (2-years-burned, n=6), surface soil that was not burned during the wildfire and collected 2 years after (2-years-unburned, n=4). The fitting error for each component is plotted as an error bar.

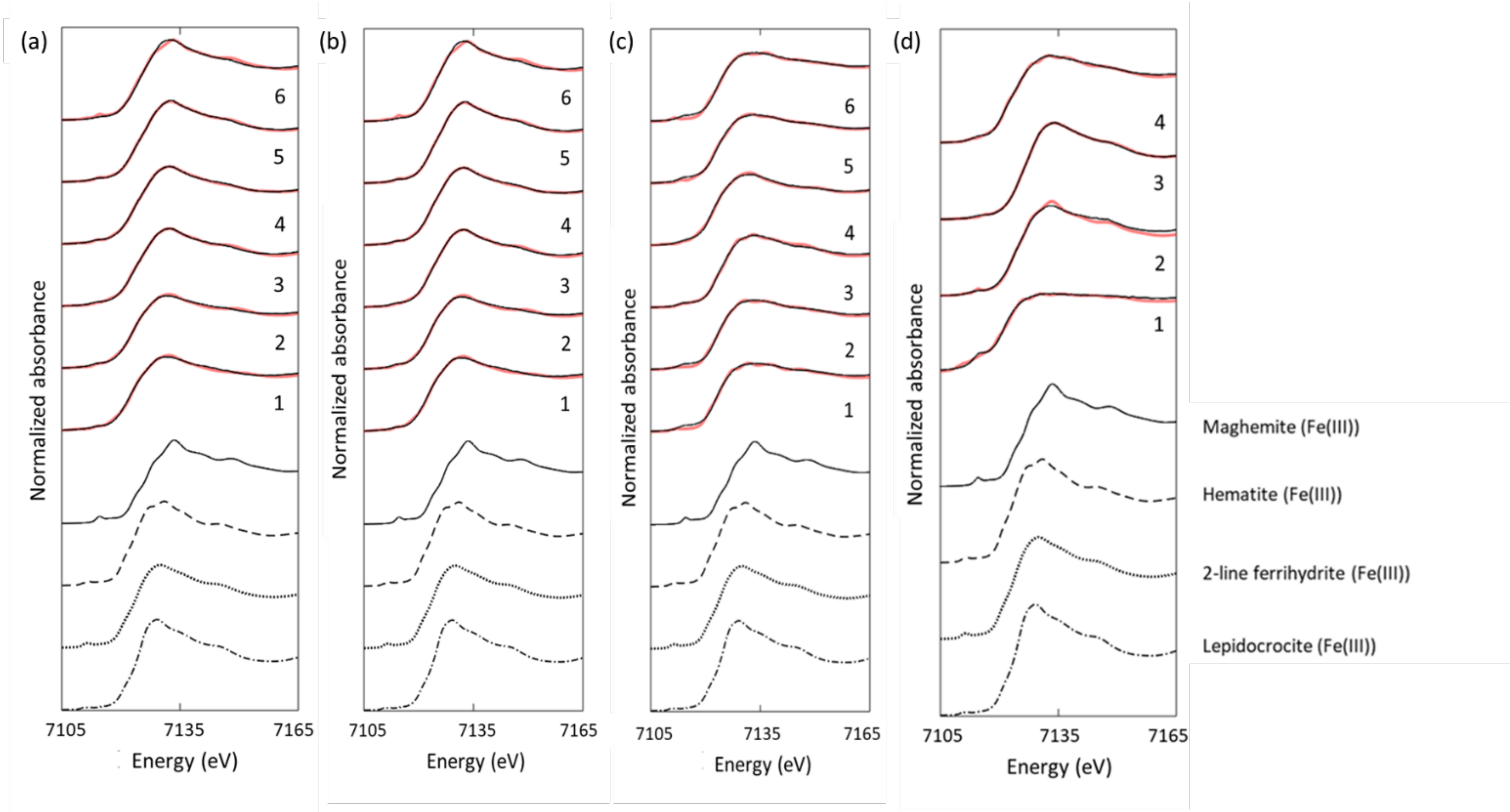

Figure S3. Iron K-edge XANES spectra were collected from different locations from (a) fir, (b) chaparral, (c) oak, and (d) pond maps shown in Figure 3. The location of XANES collected are indicated as a number. The sample spectra and the linear combination fit are shown as red and black line, respectively.

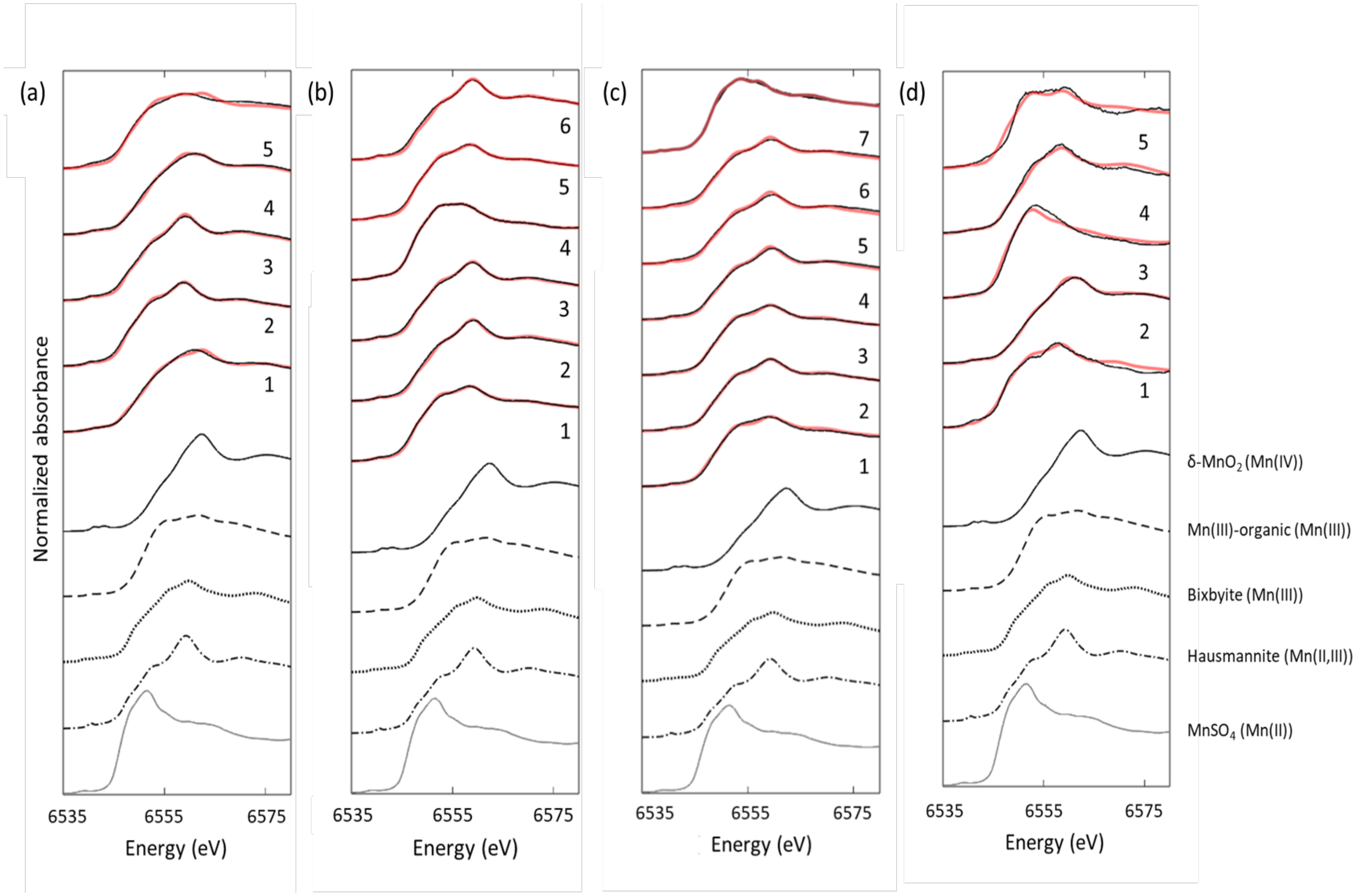

Figure S11. Manganese K-edge XANES spectra were collected from different locations from (a) fir, (b) chaparral, (c) oak, and (d) pond maps shown in Figure 4. The location of XANES collected are indicated as a number. The sample spectra and the linear combination fit are shown as red and black line, respectively.

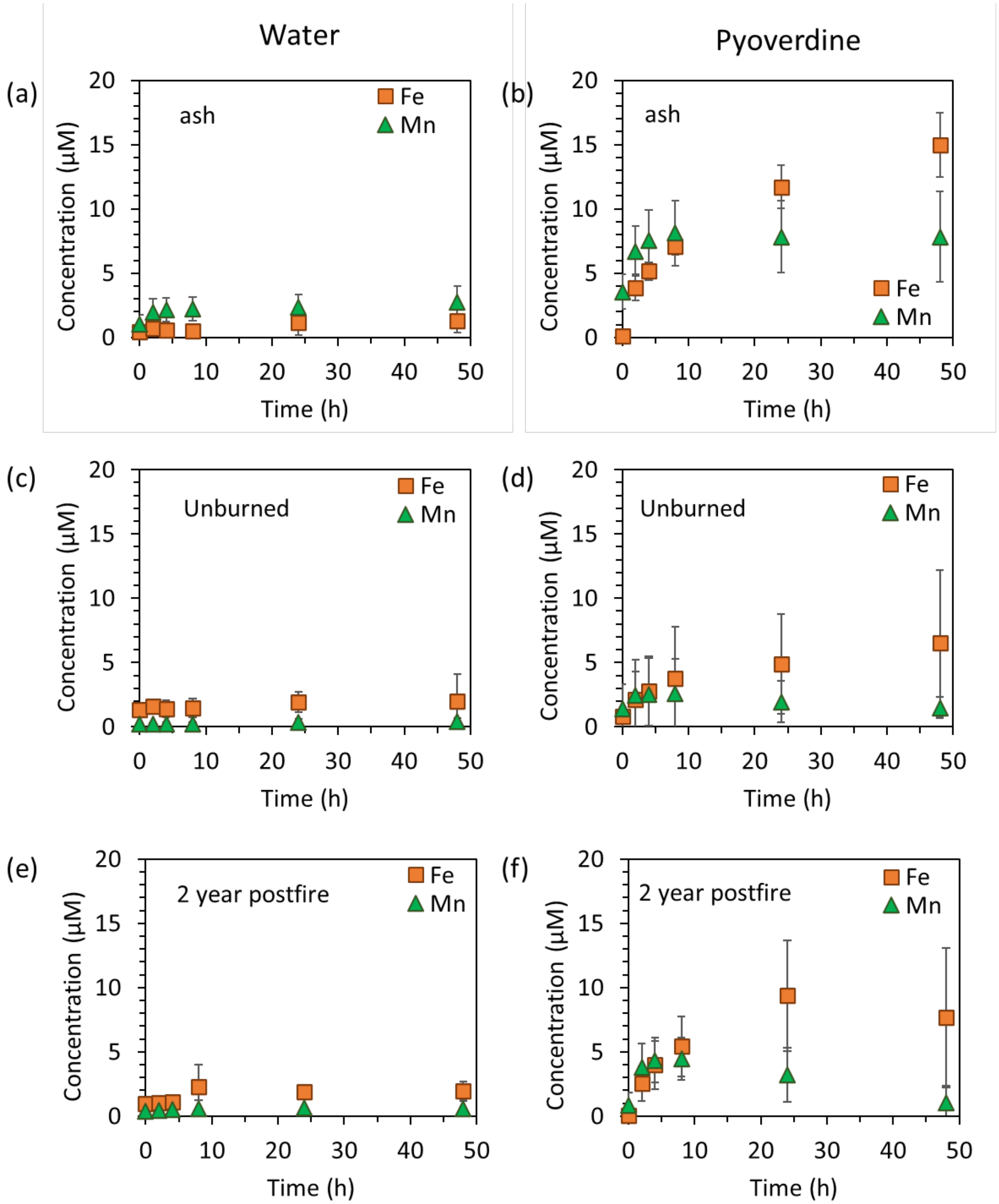

Figure S5. Fe and Mn mobilization from (a and b) ash (n=4), surface soil 2-years after the wildfire (2-years-burned, n=3, 2Y-Fir, 2Y-Chaparal-fence, 2Y-Pond), surface soil that was not burned during the wildfire and collected 2 years after (2-years-unburned, n=2, unburned Fir and Chaparral) at pH 8.0 under oxic conditions (1 g L^{-1} suspension density). The error bar represents the differences between the samples.

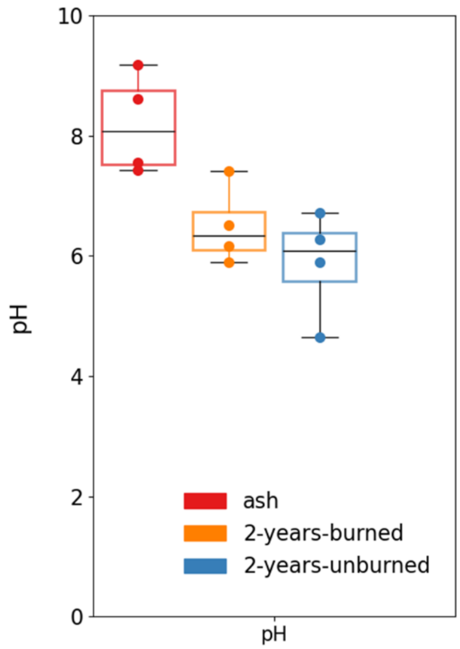

Figure S6. The pH values obtained from water extract (1:2.5, W/W) of ash, 2 year postfire surface soil and unburned soil.

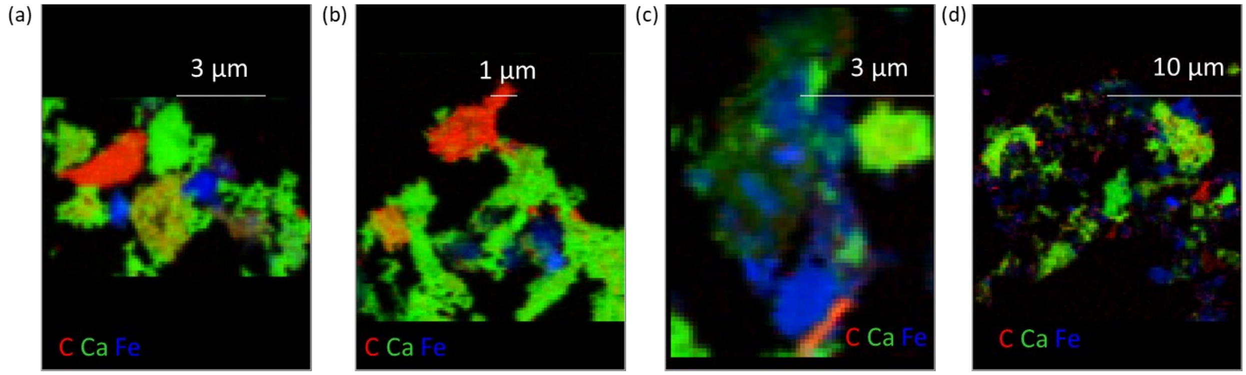

Figure S7. Tri-color maps of (a and b) chaparral and (c and d) fir samples for three STXM C/Ca/Fe NEXAFS stacks with C in red, Ca in green, and Fe in blue.

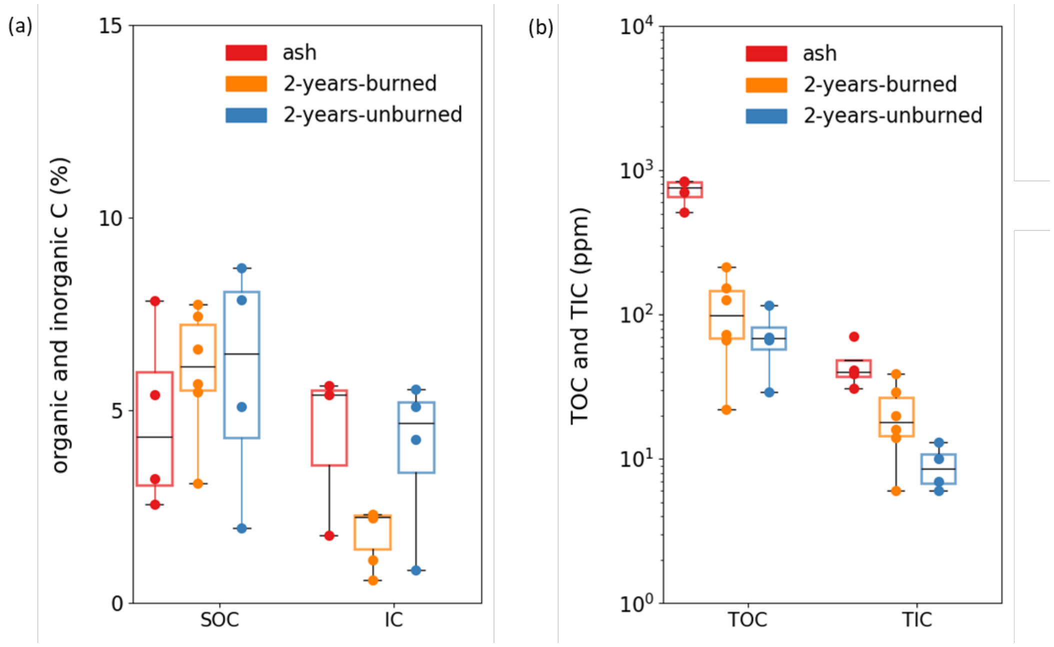

Figure S8. Total solid organic and inorganic carbon (a) of ash, 2 year postfire surface soil and unburned soil. The TOC and TIC values (b) obtained from water extract (1:2.5, W/W) of ash, 2 years postfire surface soil and unburned soil.

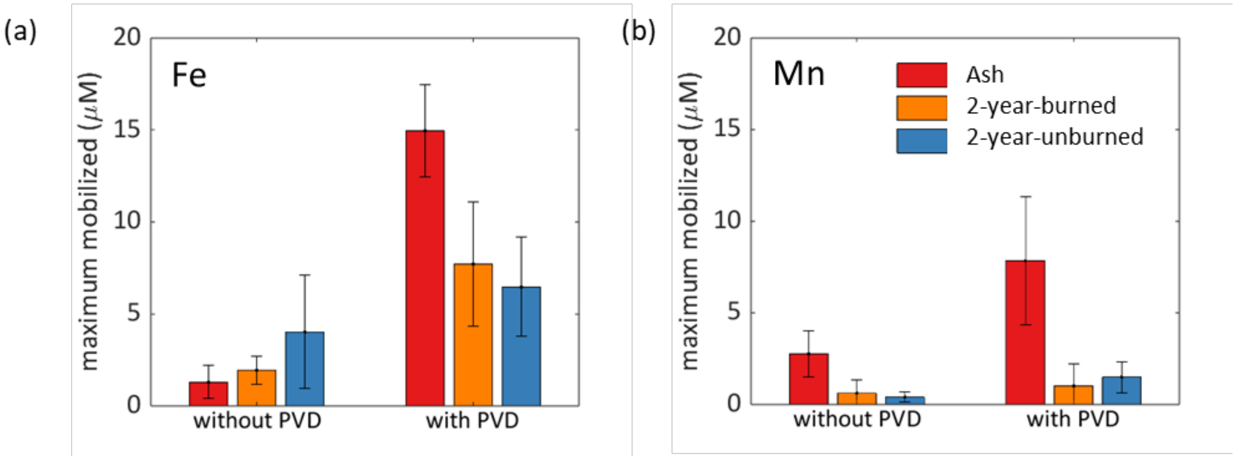

Figure S9. Maximum mobilization of Fe and Mn under oxic conditions. Bar plots show the average maximum mobilized concentrations of (a) Fe and (b) Mn from ash (n = 4, red), surface soil collected two years after the wildfire (2-year-burned, n = 3: 2Y-Fir, 2Y-Chaparral-fence, 2Y-Pond, orange), and unburned surface soil collected at the same time (2-year-unburned, n = 2: Fir and Chaparral, blue). Experiments were conducted at pH 8.0 using a 1 g L^{-1} suspension density under oxic conditions, with and without the presence of pyoverdine (PVD). Error bars represent the variability among replicate samples within each category.

References

(1) Parker, D. L.; Sposito, G.; Tebo, B. M. Manganese(III) binding to a pyoverdine siderophore produced by a manganese(II)-oxidizing bacterium. *Geochimica Et Cosmochimica Acta* **2004**, *68* (23), 4809-4820. DOI: 10.1016/j.gca.2004.05.038.
(2) National Centers for Environmental Information (NCEI). Climate Data Online (CDO). https://www.ncei.noaa.gov/cdo-web/ (accessed July 2024).
(3) Phipps, S. P. Ophiolitic olistostromes in the basal Great Valley sequence, Napa County, northern California Coast Ranges *Geological Society of America* **1984**, (198). DOI: https://doi.org/10.1130/SPE198-p103.
(4) Chen, C. M.; Dynes, J. J.; Wang, J.; Sparks, D. L. Properties of Fe-Organic Matter Associations via Coprecipitation versus Adsorption. *Environmental Science & Technology* **2014**, *48* (23), 13751-13759. DOI: 10.1021/es503669u.
(5) Prince, K. C.; Avaldi, L.; Coreno, M.; Camilloni, R.; de Simone, M. Vibrational structure of core to Rydberg state excitations of carbon dioxide and dinitrogen oxide. *Journal of Physics B-Atomic Molecular and Optical Physics* **1999**, *32* (11), 2551-2567. DOI: 10.1088/0953-4075/32/11/307.
(6) Rowley, M. C.; Nico, P. S.; Bone, S. E.; Marcus, M. A.; Pegoraro, E. F.; Castanha, C.; Kang, K. Y. L.; Bhattacharyya, A.; Torn, M. S.; Pena, J. Association between soil organic carbon and calcium in acidic grassland soils from Point Reyes National Seashore, CA. *Biogeochemistry* **2023**. DOI: 10.1007/s10533-023-01059-2.
(7) Eidenshink, J.; Schwind, B.; Brewer, K.; Zhu, Z.-L.; Quayle, B.; Howard, S. A Project for Monitoring Trends in Burn Severity. *Fire Ecology* **2007**, *3* (1), 3-21. DOI: 10.4996/fireecology.0301003.
